# Inhibition of ALKBH5 attenuates I/R-induced renal injury in male mice by promoting *Ccl28* m6A modification and increasing Treg recruitment

**DOI:** 10.1038/s41467-023-36747-y

**Published:** 2023-03-01

**Authors:** Juntao Chen, Cuidi Xu, Kun Yang, Rifeng Gao, Yirui Cao, Lifei Liang, Siyue Chen, Shihao Xu, Ruiming Rong, Jina Wang, Tongyu Zhu

**Affiliations:** 1 Department of Urology, Zhongshan Hospital, Fudan University, Shanghai, China; 2 Shanghai Key Laboratory of Organ Transplantation, Shanghai, China; 3 Shanghai Medical College Fudan University, Shanghai, China; 4 Department of Cardiology, Zhongshan Hospital, Fudan University, Shanghai Institute of Cardiovascular Diseases, Shanghai, China; 5 The Fifth People’s Hospital of Shanghai, Fudan University, Shanghai, China

**Keywords:** Acute kidney injury, Chemokines, RNA modification, Regulatory T cells

## Abstract

Ischemia reperfusion injury (IRI) is a common cause of acute kidney injury (AKI). The role of N^6-^methyladenosine (m6A) modification in AKI remains unclear. Here, we characterize the role of AlkB homolog 5 (ALKBH5) and m6A modification in an I/R-induced renal injury model in male mice. *Alkbh5*-knockout mice exhibit milder pathological damage and better renal function than wild-type mice post-IRI, whereas *Alkbh5*-knockin mice show contrary results. Also conditional knockout of *Alkbh5* in the tubular epithelial cells alleviates I/R-induced AKI and fibrosis. CCL28 is identified as a target of ALKBH5. Furthermore, *Ccl28* mRNA stability increases with *Alkbh5* deficiency, mediating by the binding of insulin-like growth factor 2 binding protein 2. Treg recruitment is upregulated and inflammatory cells are inhibited by the increased CCL28 level in IRI-*Alkbh5*^fl/fl^*Ksp*^Cre^ mice. The ALKBH5 inhibitor IOX1 exhibits protective effects against I/R-induced AKI. In summary, inhibition of ALKBH5 promotes the m6A modifications of *Ccl*28 mRNA, enhancing its stability, and regulating the Treg/inflammatory cell axis. ALKBH5 and this axis is a potential AKI treatment target.

## Abstract (web summary)

m6A modification has been reported to play roles in many developmental and pathological processes, but its role in AKI remains poorly understood. Here, the authors show the role and the mechanism of the m6A demethylase, ALKBH5 on IRI induced AKI.

## Introduction

Acute kidney injury (AKI) has emerged as a worldwide public health problem with elevating prevalence; up to 50% of critically ill patients present with AKI and it is associated with high morbidity and mortality^1–3^. Without sufficient symptomatic treatment, severe AKI can cause incomplete renal repair, persistent chronic inflammation, fibrosis progression, and eventually organ loss^4,5^. Ischemia-reperfusion injury (IRI) is a common cause of AKI. The pathophysiology of IRI, which involves hypoxia, inflammation, autophagy, apoptosis, necroptosis, oxidative stress, and mitochondrial dysfunction, is intricate and insufficiently understood^6–8^. The pathological mechanism underpinning IRI warrants further research.

Epigenetic modifications of chromatin and RNA play important roles in various biological processes and diseases^9^. Previous studies have established that kidney IRI is regulated by histone acetylation and DNA methylation^10^. Levine et al. found that inhibiting histone/protein deacetylase improved renal function and fibrosis formation^11^. Tampe et al. reported that hydralazine, a drug with potent demethylation activity, prevented fibrosis in an IRI murine model^12^. Covalent modifications of RNA nucleotides were found to regulate gene expression by affecting RNA stability and translation. N6-methyladenosine (m6A) methylation is a common type of RNA methylation that can regulate pre-mRNA splicing, 3′-end processing, nuclear export, translation, mRNA decay, and miRNA processing^13,14^.

The m6A methylation is performed by methyltransferase complexes such as methyltransferase-like protein 3 (METTL3), methyltransferase-like protein 14 (METTL14), and Wilms’ tumor 1-associating protein (WTAP)^15^. These modifications are removed by demethylases including fat mass and obesity-associated gene (FTO)^16^ and ALKBH5^17^. Reader proteins, such as YTH domain-containing proteins (YTHDFs) and insulin-like growth factor 2 binding protein (IGF2BPs) can recognize the m6A-modified RNAs^14,18^. The m6A modification has been reported to play different roles in cardiac hypertrophy; development of reproductive, hematopoietic, and central nervous systems; proliferation, differentiation, apoptosis, and migration in several cancer cells; and regulation of psychiatric disorders, metabolic syndrome, and cardiovascular disease^15^. Methyltransferases have been previously studied in renal IRI. Xu et al. found that METTL14 increased kidney injury after ischemia/reperfusion (I/R) by suppressing YAP1^19^. Another study reported that METTL3 promoted renal IRI by regulating *Foxd1* methylation^20^. However, whether m6A demethylases are involved in the pathophysiology of IRI has not been elucidated.

Here we show the dynamic change and role of ALKBH5 in I/R-induced AKI. During IRI, ALKBH5 level increases at 12 h, then decreases at 24 h and 48 h, and begins to recover at 120 h. We find that *Alkbh5* deficiency protect against I/R-induced AKI and *Alkbh5* overexpression aggravate AKI. Especially, the *Alkbh5* is mainly expressed in renal tubular epithelial cell (RTECs) and conditional knockout of *Alkbh5* in RTECs also protect against I/R-induced AKI. Mechanically, ALKBH5 regulates the m6A-mediated *Ccl28* mRNA stabilization depending on the m6A reader IGF2BP2. Furthermore, *Alkbh5* deficiency increases the recruitment of Tregs and then inhibits the inflammatory cells by increasing the secretion of CCL28. Our findings establish that *Alkbh5* deficiency protects from IRI through CCL28/Treg/inflammatory cell axis, which suggests a possible treatment approach for I/R-induced AKI.

## Results

### m6A modification and ALKBH5 were involved in the renal IRI and the hypoxia and reoxygenation (H/R) response in mRTECs

**Fig. 1:**
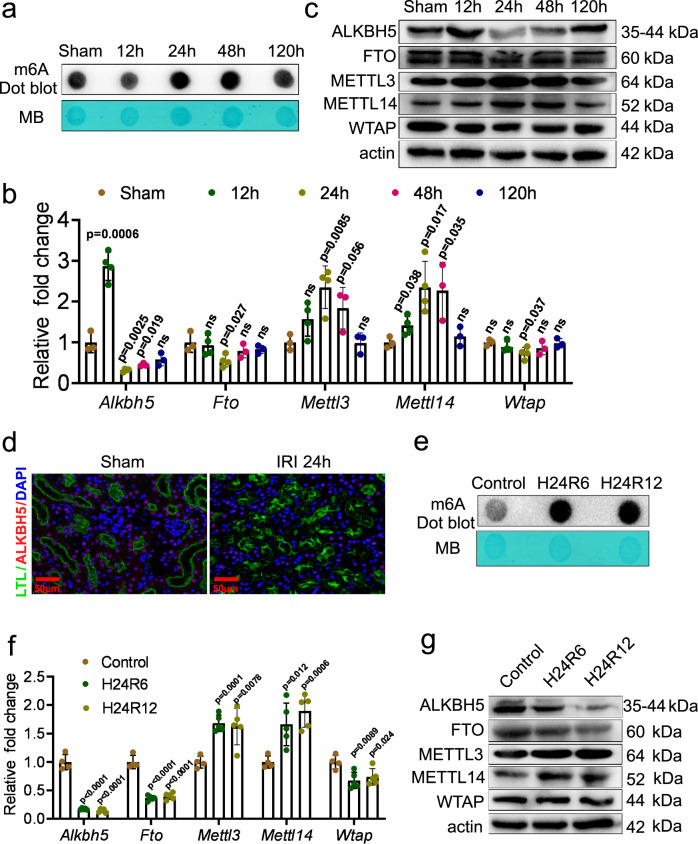
The m6A modification was involved in renal ischemia-reperfusion injury (IRI) and the hypoxia and reoxygenation (H/R) response in mouse renal tubular epithelial cells (mRTECs). **a** m6A dot blot assessed m6A mRNA methylation at different time in mouse IRI model. **b** Real-time quantitative PCR (RT-qPCR) analysis of m6A regulators: AlkB homolog 5 (*Alkbh5)*, obesity-associated gene (*Fto)*, methyltransferase-like protein 3 (*Mettl3*), methyltransferase-like protein 14 (*Mettl14*), and Wilms’ tumor 1-associating protein (*Wtap*) in IRI model. *n* = 3, 4, 4, 3, 3 for Sham, 12 h, 24 h, 48 h, and 120 h groups, respectively. Each data point represent one animal. Unpaired two-tailed Student’s t-test. **c** Western blot assay measured the protein levels of m6A regulators (ALKBH5, FTO, METTL3, METTL14, and WTAP) in IRI kidneys. Three biological repeated immunoblots have been performed. **d** Representative immunofluorescent (IF) staining of ALKBH5 (red), tetragonolobus lectin (LTL) (green), and DAPI (blue) in kidney biopsies from sham or IRI mice. (Magnification: ×400, Scale bar: 50 μm) (*n* = 5 mice for all groups). **e** m6A dot blot assessed m6A mRNA methylation of H/R model in mRTECs. **f** RT-qPCR analysis of m6A regulators (*Alkbh5*, *Fto*, *Mettl3*, *Mettl14*, and *Wtap*) in H/R model in mRTECs. *n* = 4, 5, 5 biologically independent cells for Control, H24R6, H24R12 groups, respectively. Unpaired two-tailed Student’s t-test. **g** Western blot analysis of m6A regulators (ALKBH5, FTO, METTL3, METTL14, and WTAP). Three biological repeated immunoblots have been performed. Data are showed as means ± SD.

To investigate whether m6A modification is involved in renal IRI, we detected the m6A level of RNA extracted from IRI mouse kidney tissues. The m6A level decreased initially at 12 h post-IRI, but sharply increased at 24 h and 48 h post-IRI, and then returned close to normal levels at 120 h post-IRI (Fig. 1a).

To ascertain which regulators of m6A modification function in IRI, we performed a single-cell sequencing analysis using data from the GEO database and found the demethylase *Alkbh5* was the most prominently altered gene (Fig. S1a). Then we measured the RNA expression levels of several known m6A methyltransferase- and demethylase-related proteins from kidney tissues. *Alkbh5* expression increased at 12 h, then decreased at 24 h and 48 h, and began to recover at 120 h after IRI (Fig. 1b). Changes of other regulators are shown in Fig. 1b, among which *Alkbh5* showed the most distinct changes. Western blotting (WB) analysis of these proteins demonstrated the same changes, congruent with real-time quantitative PCR (RT-qPCR) results (Fig. 1c, Fig. S1b). Immunofluorescence (IF) staining of Lotus tetragonolobus lectin (LTL) and ALKBH5 revealed that ALKBH5 was mainly expressed in RTECs and decreased at 24 h post-IRI (Fig. 1d).

H/R was applied to mRTECs. Subsequently, m6A modification and m6A methyltransferase and demethylase mRNA expression levels were measured. The results showed that the m6A modifications increased in H/R groups (both 6 h and 12 h reoxygenation) compared to the control group (Fig. 1e). The downregulation of *Alkbh5*, *Fto*, and *Wtap* mRNA and upregulation of *Mettl3*, and *Mettl14* mRNA were observed in H/R groups (Fig. 1f). The similar results were also verified by WB analysis (Fig. 1g, Fig. S1d). These data collectively showed that the expression levels of common m6A methyltransferases and demethylases, especially ALKBH5, significantly changed during renal IRI, indicating that m6A modification may play an important role in this process.

To explore which regulator mediated the downregulation of ALKBH5, we tested whether the TET enzymes altered. We found that the RNA expression of TET3 was significantly reduced after H/R but not TET1 or TET2 (Fig. S1e). When TET3 was knowdown, the expression of *Alkbh5* was significantly reduced after H/R (Fig. S1f). To further investigate the relationship between TET3 and *Alkbh5* DNA, we performed a CHIP-PCR assay using a TET3 antibody and found that TET3 could indeed bind to the *Alkbh5* promoter region (Fig. S1g). This may explain the decrease of ALKBH5 in mRTECs after H/R.

### *Alkbh5* deficiency and overexpression altered I/R-induced AKI and fibrosis

To investigate the role of ALKBH5 in renal IRI, we constructed the *Alkbh5*-knockout (KO) and *Alkbh5*-knockin (KI) mice. RT-qPCR and WB analysis showed the respective knockout and overexpression efficiency of *Alkbh5* in kidney tissue from KO (Fig. S2a and S2b) and KI mice (Fig. S2c and S2d). In addition, levels of methylases (METTL3/METTL14/WTAP) showed no significant differences after *Alkbh5* KO (Fig. S2b).

**Fig. 2:**
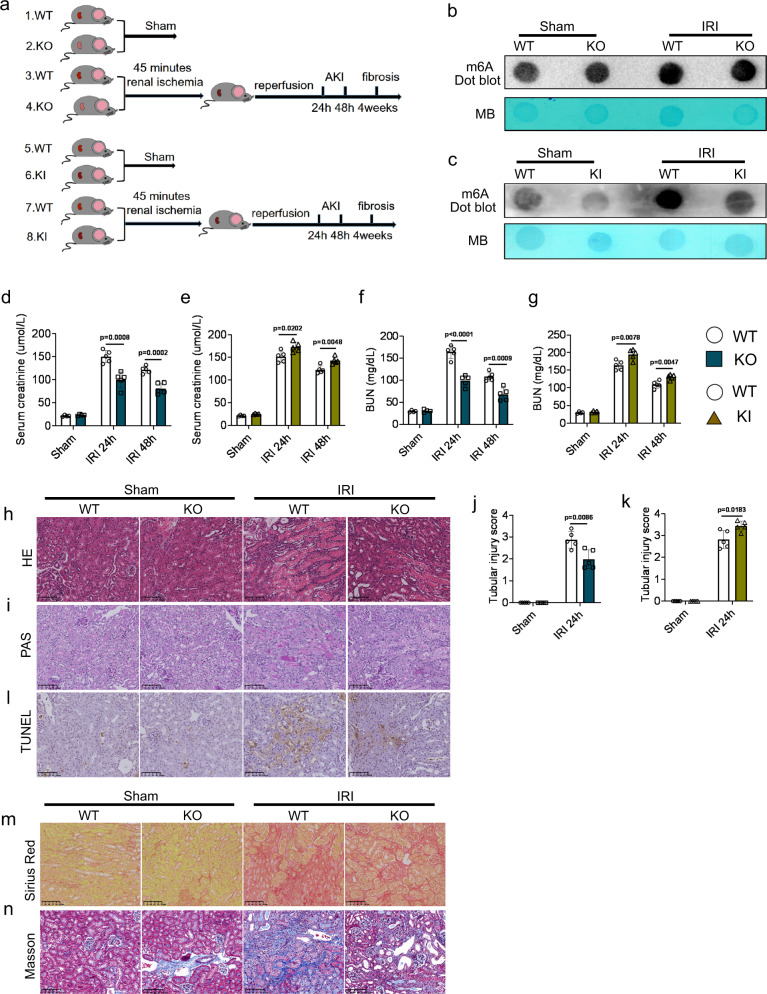
A*lkbh5* deficiency or overexpression altered I/R-induced AKI and fibrosis. **a** Schematic illustrating experiment groups. **b**, **c** m6A dot blot assessed m6A mRNA methylation of WT, KO, and KI mice in IRI model. **d**–**g** Serum creatinine and blood urea nitrogen (BUN) concentrations. **h**–**k** Representative hematoxylin and eosin (H&E), periodic acid–Schiff (PAS) staining image and tubular injury score in different groups of renal tissues. **l** The apoptosis levels in different groups were detected by terminal-deoxynucleoitidyl transferase-mediated nick end labeling (TUNEL) staining. **m**, **n** Sirius red and Masson staining were performed 4 weeks after IRI in different groups. (**h**, **i**, **l**, **m**, **n**: Magnification: ×200, Scale bar: 100 μm) (**h**–**n**: *n* = 5 for all groups). For **d**–**g**, Sham groups, *n* = 4; IRI 24 h, 48 h groups, *n* = 5. Each data point represent one animal. Unpaired two-tailed Student’s t-test. Data are showed as means ± SD.

We subsequently performed unilateral renal pedicle clamping for 45 min or sham surgery in WT, KO, and KI mice (Fig. 2a). The m6A methylation in the IRI-KO and IRI-KI groups were respectively higher and lower than that of the IRI-WT group (Fig. 2b, c). Among the Sham-WT, Sham-KO, and Sham-KI groups, there were no significant differences, either in renal function assays including: serum creatinine and blood urea nitrogen (BUN) (Fig. 2d–g) or in the pathological score analyses (Fig. 2h–k). However, in IRI groups, KO mice exhibited better renal function (lower serum creatinine and BUN; Fig. 2d, f), milder renal damage (revealed with hematoxylin and eosin [H&E] and periodic acid–Schiff [PAS] staining; Fig. 2h–j), and less renal cell apoptosis (Fig. 2l) compared to WT mice. Correspondingly, IRI-KI mice exhibited worse renal function (higher serum creatinine and BUN; Fig. 2e, g), more severe renal damage (revealed with H&E and PAS staining; Fig. 2k, Fig. S3a and S3b), and more renal cell apoptosis (Fig. S3c) compared to IRI-WT mice.

Sirius red and Masson staining were performed to detect the severity of renal fibrosis 4 weeks post-IRI, showing that IRI-KO and IRI-KI mice respectively exhibited fewer and more positive staining points than IRI-WT mice (Fig. 2m, n) (Fig. S3d and S3e). Our data showed that *Alkbh5* deficiency protected against I/R-induced AKI and fibrosis. Conversely, *Alkbh5* overexpression aggravated kidney injury after I/R.

### *Alkbh5* deficiency in RTECs protected I/R-induced AKI and fibrosis

**Fig. 3:**
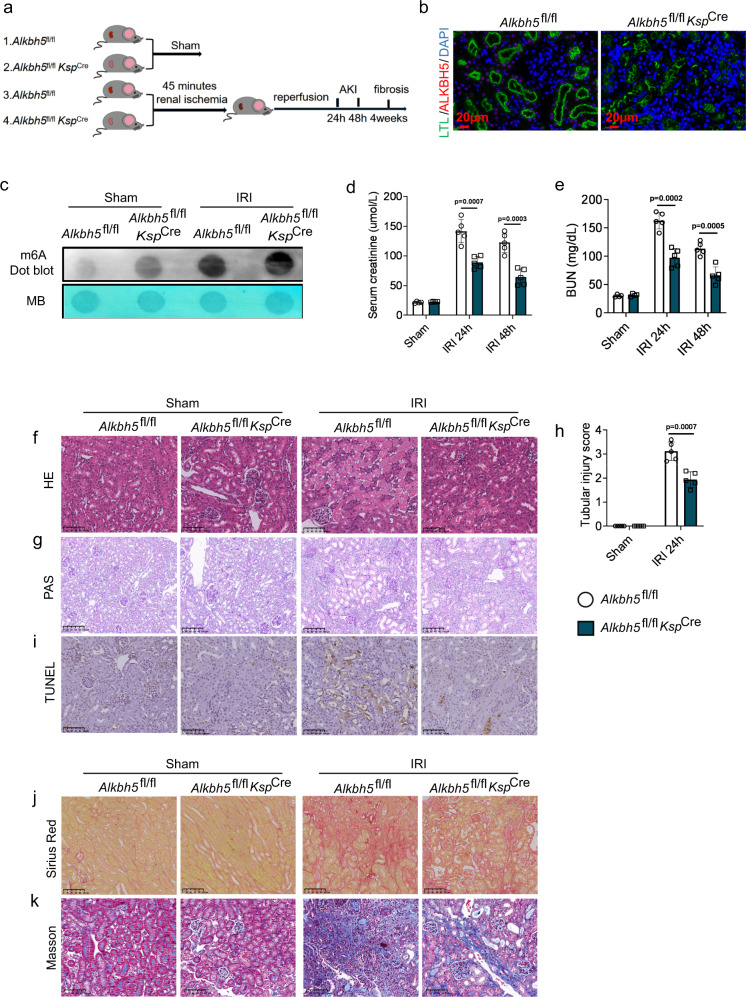
*Alkbh5* deficiency in RTECs protected I/R-induced AKI and fibrosis. **a** Schematic illustrating experiment groups and treatment in 4 groups. **b** Representative IF staining of ALKBH5 (red), LTL (green), and DAPI (blue) in kidney biopsies from *Alkbh5*^fl/fl^ and *Alkbh5*^fl/fl^*Ksp*^Cre^ mice. (Magnification: ×400, Scale bar: 20 μm) (*n* = 5 for all groups). **c** m6A dot blot assessed m6A mRNA methylation of *Alkbh5*^fl/fl^ and *Alkbh5*^fl/fl^*Ksp*^Cre^mice in IRI model. **d**, **e** Serum creatinine and BUN concentrations. **f**–**h** Representative H&E and PAS staining image and tubular injury score in different groups of renal tissues. **i** The apoptosis levels in different groups were detected by TUNEL staining. **j**, **k** Sirius red and Masson staining were performed 4 weeks after IRI in different groups. (**f**–**j**: Magnification: ×200, Scale bar: 100 μm) (**i**–**k**: *n* = 4 for sham groups, *n* = 5 for IRI groups). For **d**, **e**, Sham groups, *n* = 4; IRI 24 h, 48 h groups, *n* = 5. For **h**, *n* = 5 for all groups. Each data point represent one animal, with unpaired Student’s t-test performed. Data are shown as means ± SD. Unpaired two-tailed Student’s t-test.

To investigate whether the loss of *Alkbh5* in RTECs exerts a protective effect in renal IRI, we constructed *Alkbh5*-cKO mice by crossing *Alkbh5*^fl/fl^ mice with *Ksp*^Cre^ mice (Fig. S4a). All mice were genotyped by PCR (Fig. S4b). The absence of *Alkbh5* in RTECs was verified by RT-qPCR (Fig. S4c), WB (Fig. S4d), and immunofluorescence (IF) staining (Fig. 3b). We subsequently induced IRI in *Alkbh5*^fl/fl^ and *Alkbh5*^fl/fl^*Ksp*^Cre^ mice (Fig. 3a). The m6A dot blot revealed that m6A methylation further increased in the IRI-*Alkbh5*^fl/fl^*Ksp*^Cre^ group rather than IRI-*Alkbh5*^fl/fl^ group (Fig. 3c). There were no significant differences in serum creatinine and BUN levels between *Alkbh5*^fl/fl^ and *Alkbh5*^fl/fl^*Ksp*^Cre^ mice who underwent sham surgery (Fig. 3d, e). However, the serum creatinine and BUN in the IRI-*Alkbh5*^fl/fl^*Ksp*^Cre^ group were all significantly lower than those in IRI-*Alkbh5*^fl/fl^ group both 24 h and 48 h after IRI (Fig. 3d, e).

Next, we harvested the kidney tissues from four groups at 24 h post-IRI for pathologic analysis. H&E and PAS staining showed that *Alkbh5* deficiency in RTECs attenuated renal injury post I/R (Fig. 3f–h). In addition, TUNEL staining also showed *Alkbh5* cKO could reduce cell apoptosis (Fig. 3i). These data showed that deletion of *Alkbh5* in RTECs protected against I/R-induced AKI. To further determine the function of *Alkbh5* deficiency in chronic kidney injury 4 weeks post I/R, Sirius red and Masson staining were performed, showing that the IRI-*Alkbh5*^fl/fl^*Ksp*^Cre^ group exhibited milder fibrosis than the IRI-*Alkbh5*^fl/fl^ group (Fig. 3j, k). Collectively, *Alkbh5* deficiency in RTECs effectively protected against AKI and chronic fibrosis after I/R.

### Characterization of m6A modification and gene expression changes in *Alkbh5*-KO mice

**Fig. 4:**
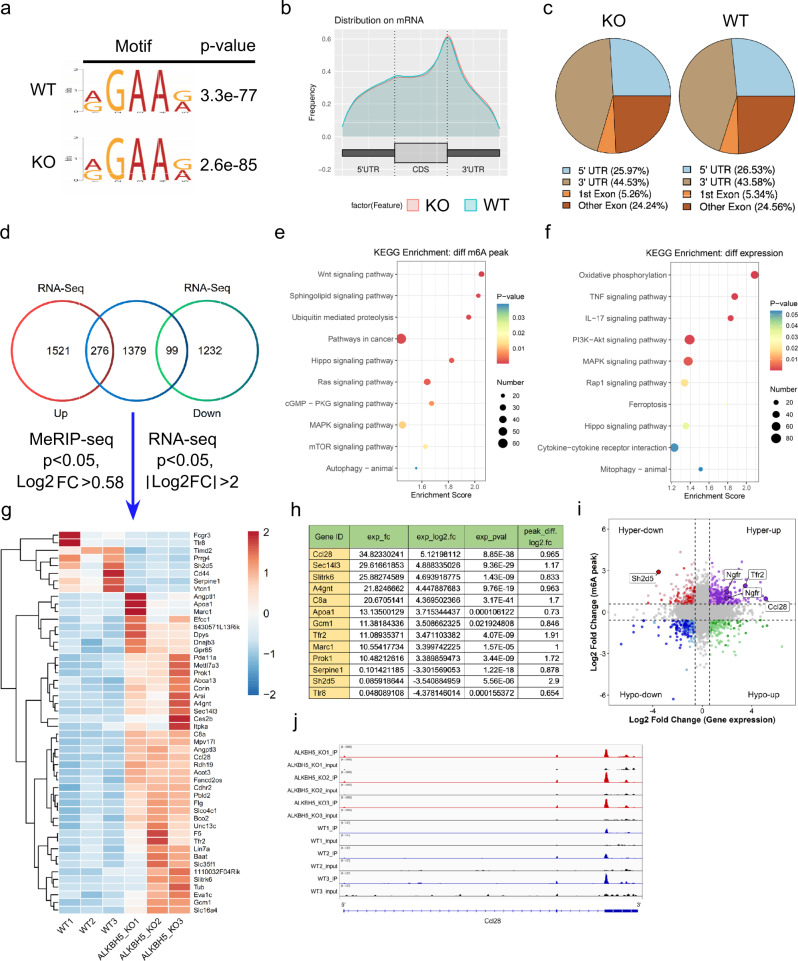
Characterization of m6A modification and gene expression changes between the *Alkbh5-*KO and WT mice. **a** Predominant motif identified with m6A-seq peaks in IRI-WT group or IRI-KO group. Motif enrichment *p*-value was calculated based on Fisher’s Exact Test. **b** Distribution of m6A peaks across mRNA in IRI-WT group and IRI-KO group. **c** Pie graphs of m6A peak distribution in indicated regions in IRI-WT group and IRI-KO group. **d** Venn diagrams show 276 upregulated expression genes and 99 downregulated expression genes with m6A peak change in IRI-KO group compared with IRI-WT group. **e**, **f** Kyoto Encyclopedia of Genes and Genomes (KEGG) enrichment analysis correlated with different expression genes and different m6A peak genes. KEGG enrichment *p*-value was calculated based on the hypergeometric distribution. **g** Heatmap of RNA-seq analysis showing top differentially expressed genes (MeRIP-seq *p* < 0.05, Log2 FC > 0.58 and RNA-seq *p* < 0.05, |Log2 FC| > 2). **h** The detailed information of the changed genes with the expression fold change (exp_fc) >10 or <0.1. Differential expression analysis was performed using the DESeq2 R package based on negative binomial distribution model. **i** Four-quadrant scatter plot showed the distribution of genes with both differential (hyper or hypo) m6A peaks (Y axis) and differential (up or down) expression (X axis) in IRI-KO group compared with IRI-WT group. **j** Deficiency of *Alkbh5* increased m6A modification of *Ccl28* mRNA in IRI-KO group.

To identify the direct m6A demethylation targets of ALKBH5 at the onset of renal IRI, we performed methylated RNA immunoprecipitation sequencing (MeRIP-seq) and RNA sequencing (RNA-seq) using RNA isolated from IRI mouse kidneys of WT and KO mice 24 h after I/R (WT *n* = 3 vs. KO *n* = 3). According to the MeRIP-seq analysis, m6A modifications were typically located in a consensus “RGAAR” motif (R = A or G) (Fig. 4a), which has not been previously reported. m6A peaks were particularly abundant around stop codons (Fig. 4b, c). MeRIP-seq analysis identified 1754 hypermethylated peaks in the KO group. RNA-seq revealed that 1779 genes were upregulated and 1331 genes were downregulated in the KO group compared to the WT group (Fig. 4d). The heatmap of differentially expressed genes is shown in Fig. S5a. Gene Ontology (GO) enrichment analysis using RNA-seq (including long noncoding RNA and circular RNA) and MeRIP-seq data revealed a common subset of transcripts that were largely associated with metabolism, oxidative stress, and immunity such as the oxidation-reduction process, metabolic process, mitochondrion, immune system process, and neutrophil chemotaxis (Fig. S5b–d). The same data were BLASTed and mapped to pathways in the Kyoto Encyclopedia of Genes and Genomes (KEGG), revealing a variety of signaling pathways related to immunity and metabolism, such as cytokine-cytokine receptor interaction, TNF signaling, IL-17 signaling, TGF-beta signaling, oxidative phosphorylation, PI3K/Akt signaling, mTOR signaling, and autophagy (Fig. 4e, f and Fig. S5e).

To further evaluate the mRNA expression and m6A methylation levels of the corresponding genes, we selected the top differentially expressed genes (Fig. 4g) and listed the details of the altered genes with expression fold changes (exp_fc) >10 or <0.1 (Fig. 4h). Furthermore, we used a four-quadrant scatter plot for an in-depth data analysis of the four distinct subgroups and their functions (Fig. 4i). To find the most possible direct target genes of ALKBH5, we selected the most up- or down-regulated genes associated with hypermethylation, such as *Ccl28*, *Sh2d5*, *Ngfr*, and *Tfr2* (Fig. 4i). In the MeRIP-seq data, we found one m6A peak around the stop codon of *Ccl28* mRNA in the KO group, which was diminished in the WT group (Fig. 4j). The m6A peaks of other genes are also shown in Supplementary Fig. 5f–h. *Ccl28* was the significantly differentially expressed gene with the highest expression fold change, so we hypothesized *Ccl28* as the direct target of ALKBH5.

### CCL28 is a direct target of ALKBH5

**Fig. 5:**
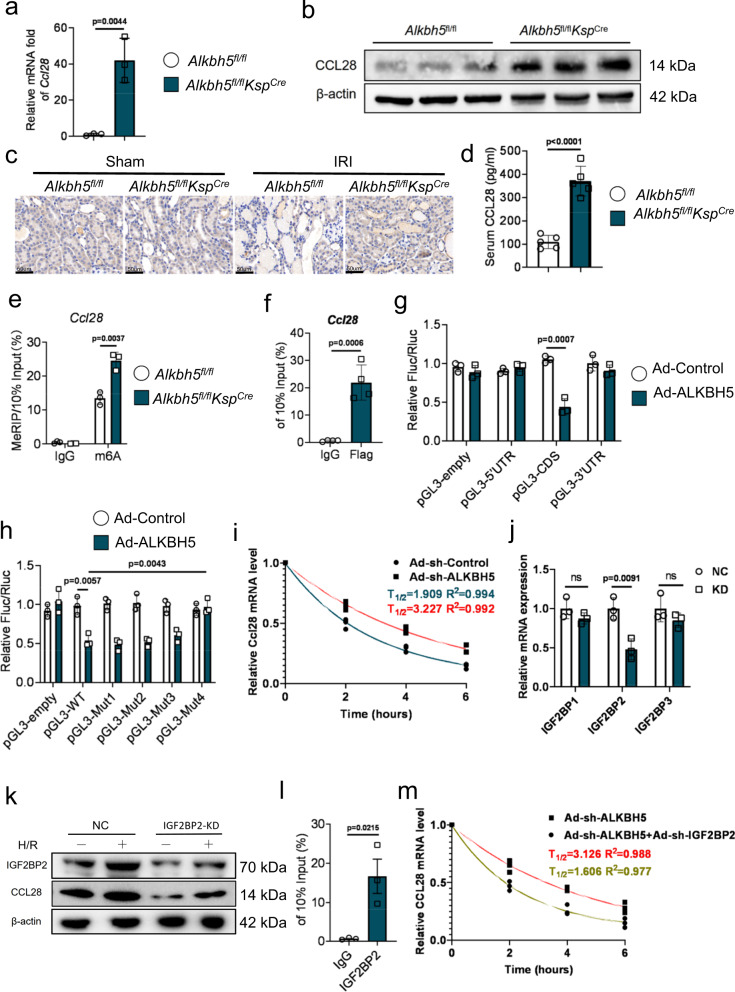
ALKBH5 enhances *Ccl28* mRNA stability in a m6A-dependent manner. **a** RNA level of *Ccl28* in IRI-*Alkbh5*^fl/fl^*Ksp*^Cre^ group and IRI-*Alkbh5*^fl/fl^ group was examined by RT-qPCR. *n* = 3 biologically independent animals. **b** Protein level of CCL28 in IRI-*Alkbh5*^fl/fl^*Ksp*^Cre^ group and IRI-*Alkbh5*^fl/fl^ group was examined by western blot. **c** Representative IHC staining data of CCL28 in kidney biopsies from IRI mice. (Magnification: ×400, Scale bar: 40 μm). **d** Serum CCL28 level was examined by ELISA. *n* = 5 biologically independent animals. **e** m6A modification of *Ccl28* mRNA was detected by MeRIP-qPCR analysis using anti-IgG and anti-m6A antibodies. *n* = 3 biologically independent animals. **f** Relative enrichment of *Ccl28* mRNA associated with ALKBH5 protein was identified by RIP assays using anti-IgG and anti-FLAG antibodies. *n* = 4 biologically independent experiments. **g** Relative activity of the pGL3-empty, pGL3-CDS, pGL3-5’UTR, and pGL3-3’UTR luciferase reporter in Ad-Control and Ad-ALKBH5 groups. *n* = 3 biologically independent experiments. **h** Relative activity of the pGL3-empty, pGL3-WT, pGLMut1, pGLMut2, pGLMut3, and pGLMut4 luciferase reporter in Ad-Control and Ad-ALKBH5 groups. *n* = 3 biologically independent experiments. **i** ALKBH5-silenced and control cells were treated with actinomycin D and harvested at 0, 2, 4, and 6 h. RNA decay rate was determined to estimate the stability of *Ccl28* mRNA (normalized to the expression at 0 h). *n* = 3 biologically independent experiments. **j** RT-qPCR analysis of *Ccl28* in mRTECs after different insulin-like growth factor 2 binding proteins (IGF2BPs) knockdown compared to NC treatment. *n* = 3 biologically independent experiments. **k** Western blot analysis of CCL28 after IGF2BP2 knockdown in H/R-treated mRTECs. Three biological repeated immunoblots have been performed. **l** RT-qPCR analysis of RIP assays in H/R-treated mRTECs showing the direct binding between the IGF2BP2 protein and *Ccl28* mRNA. *n* = 3 biologically independent experiments. **m** ALKBH5-silenced with/without IGF2BP2-silenced cells were treated with actinomycin D and harvested at 0, 2, 4, and 6 h. RNA decay rate was determined to estimate the stability of *Ccl28* mRNA (normalized to the expression at 0 h). *n* = 3 biologically independent experiments. Data are shown as means ± SD. Unpaired two-tailed Student’s t-test for (**a**), (**d**–**h**), (**j**), (**l**).

The expression of *Ccl28* in the IRI-*Alkbh5*^fl/fl^*Ksp*^Cre^ and IRI-*Alkbh5*^fl/fl^ groups were detected by RT-qPCR, WB and immunohistochemistry (IHC). The results showed that the mRNA and protein of CCL28 were more abundant in *Alkbh5*^fl/fl^*Ksp*^Cre^ mice than IRI-*Alkbh5*^fl/fl^ mice (Fig. 5a–c, Fig. S6a). Similar changes of CCL28 were also observed in serum by ELISA (Fig. 5d). Then, MeRIP was performed using anti-m6A antibody or IgG antibody. Immunoprecipitation with the anti-m6A antibody resulted in a highly enriched *Ccl28* mRNA level compared with those obtained with the IgG antibody (Fig. 5e). These results revealed that m6A modification was involved in the regulation of *Ccl28* mRNA by ALKBH5.

To determine whether there is a direct interaction of ALKBH5 and *Ccl28* mRNA, we performed RNA-IP using an adenoviral flag-tagged ALKBH5 vector (Ad-ALKBH5) (Fig. S6b). Immunoprecipitation with the anti-Flag antibody group revealed higher levels of *Ccl28* mRNA compared to those obtained with the IgG antibody (Fig. 5f).

We subsequently screened the possible ALKBH5-mediated m6A modification sites in *Ccl28* mRNA by combining data from MeRIP-seq and the RMBase database, finding potential m6A-modified sites located in CDS, which is close to the terminator region (Fig. S6c). We constructed luciferase reporter vectors respectively for the 5′UTR, CDS, and 3′UTR (Fig. S6d). The result showed that ALKBH5 overexpression decreased the luciferase activity of the pGL3-CDS, but not the pGL3-5′UTR or pGL3-3′UTR (Fig. 5g). We then further constructed the *Ccl28* mRNA coding sequence (pGL3-CCL28-WT) and mutated sequence (pGL3-CCL28-Mut1,2,3,4) luciferase reporter vectors to find the direct m6A sites (Fig. S6e). ALKBH5 overexpression decreased luciferase activity of pGL3-CCL28-WT and pGL3-CCL28-Mut1,2,3, but did not affect pGL3-CCL28-Mut4 (Fig. 5h). This demonstrated that the potential m6A site (Mut4) on *Ccl28* mRNA is an ALKBH5-mediated demethylation site.

A previous study reported that ALKBH5 disrupted mRNA stability by m6A demethylation^21^, so we hypothesized ALKBH5 deficiency stabilized *Ccl28* mRNA. The *Ccl28* mRNA levels were tested at 0, 2, 4, and 6 h after inhibiting RNA polymerase via actinomycin D treatment in mRTECs. RT-qPCR revealed the half-life of *Ccl28* was increased after ALKBH5 knockdown, suggesting that decreased ALKBH5 stabilized *Ccl28* mRNA (Fig. 5i). IGF2BPs are m6A readers with proven mRNA stability-enhancing effects. Therefore, we detected whether the IGF2BPs (IGF2BP1, IGF2BP2, IGF2BP3) are involved in the regulation of *Ccl28* mRNA stability. When IGF2BP2 was knocked down, *Ccl28* mRNA expression was markedly suppressed. But knockdown of IGF2BP1 or IGF2BP3 had a limited effect (Fig. 5j). WB analysis also indicated that IGF2BP2 knockdown inhibited CCL28 protein abundance (Fig. 5k, Fig. S6f). An RNA immunoprecipitation (RIP) analysis using an anti-IGF2BP2 antibody further confirmed the interaction between IGF2BP2 and *Ccl28* mRNA in H/R mRTECs (Fig. 5l). An RNA stability assay showed that IGF2BP2 knockdown reversed the stability-enhancing effects on *Ccl28* mRNA of ALKBH5 knockdown (Fig. 5m). Therefore, we identified IGF2BP2 as the reader protein in the regulation of *Ccl28* mRNA stability.

### *Alkbh5* deficiency increased the CCL28-mediated recruitment of Tregs

**Fig. 6:**
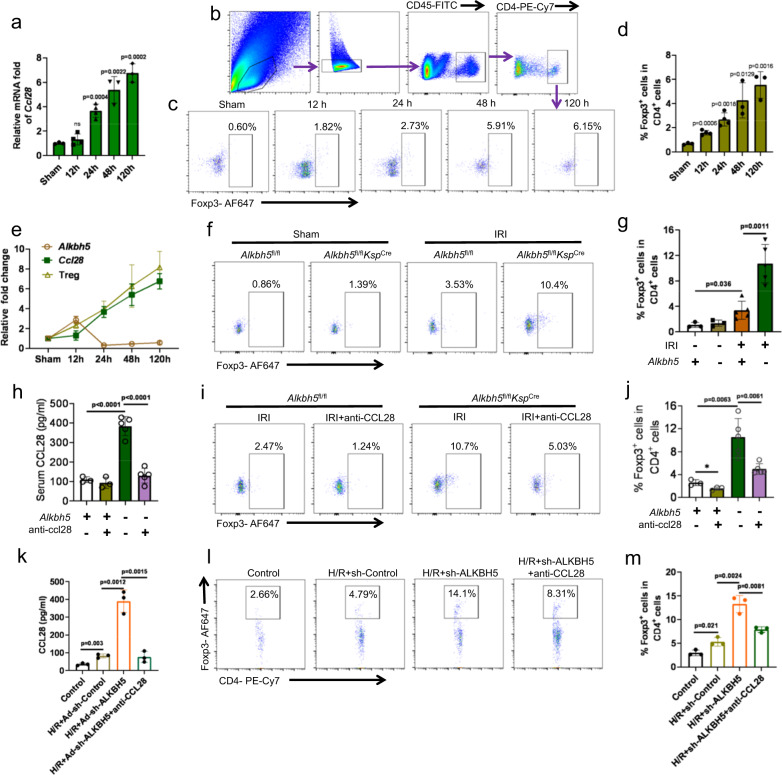
A*lkbh5* deficiency increased the recruitment of Tregs through CCL28. **a** RNA level of *Ccl28* in different times after I/R was examined by RT-qPCR. *n* = 3, 4, 4, 3, 3 biologically independent animals for Sham, 12 h, 24 h, 48 h, and 120 h groups, respectively. **b** The FACS analysis process of Tregs. **c**, **d** Representative flow cytometry dot plots and percentage of CD4^+^Foxp3^+^ cells among the CD4^+^ T cells at 0 h, 12 h, 24 h, 48 h, and 120 h after I/R. *n* = 3, 4, 4, 3, 3 biologically independent animals for Sham, 12 h, 24 h, 48 h, and 120 h groups, respectively. **e** The line chart showed the relationship of ALKBH5, CCL28, and Tregs. **f**, **g** Renal recruitment of CD4^+^Foxp3^+^ cells 24 h after IRI in 4 groups. *n* = 3 for Sham groups, *n* = 5 for IRI groups. Each data point represents one animal. **h** The CCL28 protein in serum from IRI-*Alkbh5*^fl/fl^*Ksp*^Cre^ or IRI-*Alkbh5*^fl/fl^ mice with or without CCL28 antibody treatment. **i**, **j** Representative flow cytometry dot plots and percentage of CD4^+^Foxp3^+^ cells among the CD4^+^ T cells in IRI-*Alkbh5*^fl/fl^*Ksp*^Cre^ or IRI-*Alkbh5*^fl/fl^ mice with or without CCL28 antibody treatment. *n* = 3 for IRI-*Alkbh5*^fl/fl^ groups, *n* = 5 for *Alkbh5*^fl/fl^*Ksp*^Cre^ groups. Each data point represents one animal. **k** CCL28 protein in supernatants from mRTECs cells incubated under hypoxic or oxic conditions with or without Ad-sh-ALKBH5 and CCL28 antibody treatment, as determined by ELISA. *n* = 3 biologically independent experiments. **l**, **m** Representative flow cytometry dot plots and percentage of recruited CD4^+^Foxp3^+^ cells among the CD4^+^ T cells in groups with or without hypoxic, Ad-sh-ALKBH5, and CCL28 antibody treatment. *n* = 3 biologically independent experiments. Data are shown as means ± SD. Unpaired two-tailed Student’s t-test for (**a**), (**d**), (**e**), (**g**), (**h**), (**j**), (**k**), and (**m**).

To identify the function of CCL28 in AKI, we detected the *Ccl28* expression level in kidney tissues at different time points after IRI. The results showed that *Ccl28* expression increased after IRI and peaked at 120 h (Fig. 6a). In addition, we searched for *Ccl28* in an online analyzer for kidney single-cell datasets. *Ccl28*, which was mainly expressed in PT (proximal tubule) cells especially PTS3 and increased after IRI, reaching an expression peak at six weeks in a single-cell sequencing experiment investigating mouse IRI kidneys^22^ (Fig. S7a). A similar result was shown in a spatial transcriptomics investigation of female mouse IRI kidneys^23^ (Fig. S7b).

Subsequently, we induced I/R in *Ccl28*-KO and WT mice. The results showed that serum creatinine and BUN were significantly higher in the *Ccl28*-KO group after I/R than in the WT group (Fig. S8a, S8b). H&E staining demonstrated that *Ccl28* knockout aggravated I/R-induced renal injury (Fig. S8c, S8d). CCL28 expression was reportedly induced by tumor cells under hypoxia, and could recruit Tregs^24^. Further investigation indicated that *Ccl28* knockout could decrease infiltrated Treg levels (Fig. S8e and S8f) and increase infiltrated macrophage and neutrophil levels in the kidney post-IRI (Fig. S8g). These data indicated that CCL28 could increase the Tregs, inhibit the inflammatory cells, and finally attenuate the I/R-induced AKI.

Therefore, we detected the infiltration of Tregs in kidney tissues at various time points after IRI. The number of Tregs changed consistently with *Ccl28* expression (Fig. 6b–d). Together, we concluded that the levels of *Ccl28* and Tregs increased following the decrease of *Alkbh5* (Fig. 6e). We hypothesized that the recruitment of Tregs is involved in the mechanism by which *Alkbh5* deficiency and CCL28 upregulation induce protective effects in IRI. We compared the percentage of Tregs in kidney tissues 24 h after IRI in different groups. The infiltration of Tregs in the IRI-*Alkbh5*^fl/fl^*Ksp*^Cre^ group was significantly higher than that in IRI-*Alkbh5*^fl/fl^ group (Fig. 6f, g).

To further explore whether CCL28 was indeed a key factor in recruiting Tregs, we administered an anti-CCL28 antibody to the different groups of mice (Fig. 6h). Following anti-CCL28 antibody treatment, the percentage of Tregs was significantly decreased in *Alkbh5*^fl/fl^*Ksp*^Cre^ mice after IRI compared with *Alkbh5*^fl/fl^*Ksp*^Cre^ mice without antibody treatment (Fig. 6i, j).

We then tested the chemotactic activity towards freshly isolated mouse peripheral blood mononuclear cells (PBMCs) using an in vitro transwell assay. The CCL28 level was significantly increased in the H/R + Ad-sh-ALKBH5 group, and was reversed using an anti-CCL28 antibody in the H/R + Ad-sh-ALKBH5 + anti-CCL28 group (Fig. 6k). We observed an increasing percentage of Foxp3^+^CD4^+^ cells among CD4^+^ cells in the H/R + Ad-sh-ALKBH5 group and a significant decrease after CCL28 antibody administration (Fig. 6l, m).

### The CCL28/Treg/inflammatory cell axis is involved in the mechanism underlying the therapeutic effect of *Alkbh5* deficiency in I/R-induced AKI

**Fig. 7:**
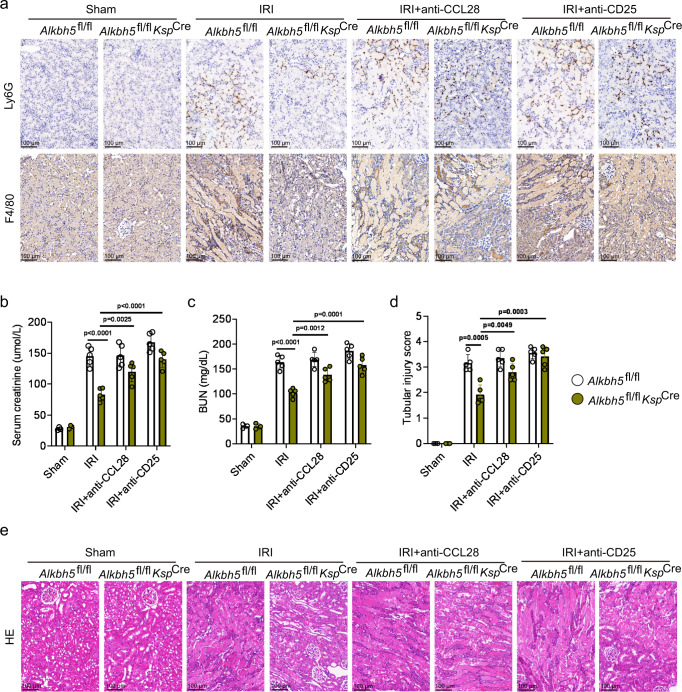
CCL28/Treg/inflammatory cells axis is involved in mechanism of therapeutical effect of *Alkbh5* deficiency on I/R-induced AKI. **a** Infiltration of Ly6G^+^ neutrophil and F4/80^+^ macrophages in different groups of renal tissues. **b**, **c** Serum creatinine and BUN concentrations. **d**, **e** Tubular injury score and representative H&E staining image in different groups of renal tissues. (**a**, **e**: Magnification: ×200, Scale bar: 100 μm). For **b**–**d**, Sham groups, n = 3; other groups, n = 5. Each data point represents one animal. Unpaired two-tailed Student’s t-test. Data are shown as means ± SD.

A previous study reported that Tregs suppressed neutrophils, macrophages, and the innate immune system in kidney IRI^25^. Our results showed that IRI kidney tissues from *Alkbh5*^fl/fl^*Ksp*^Cre^ mice showed fewer Ly6G^+^ neutrophils and F4/80^+^ macrophages compared to *Alkbh5*^fl/fl^ mice (Fig. 7a).

To demonstrate the function of the CCL28/Treg/inflammatory cell axis, CCL28 and CD25 antibodies were administered to *Alkbh5*^fl/fl^*Ksp*^Cre^ or *Alkbh5*^fl/fl^ mice. The IHC staining targeting Ly6G and F4/80 showed that CCL28 or CD25 antibody administration significantly increased the numbers of neutrophils and macrophages in *Alkbh5*^fl/fl^*Ksp*^Cre^ mice compared to those without CCL28 or CD25 antibody administration (Fig. 7a). This result revealed that ALKBH5 regulates inflammation after IRI through CCL28-mediated Treg recruitment. The renal function and pathology analysis showed that CCL28 or CD25 antibody treatment reversed the protective effect of *Alkbh5* deficiency (Fig. 7b–e). In summary, CCL28 and Treg blockade effectively inhibited the protective effect of *Alkbh5* knockout. The CCL28/Treg/inflammatory cell axis is a direct downstream target of ALKBH5 in IRI.

### The ALKBH5 inhibitor IOX1 and recombinant mouse CCL28 exert protective effects in vivo during IRI

**Fig. 8:**
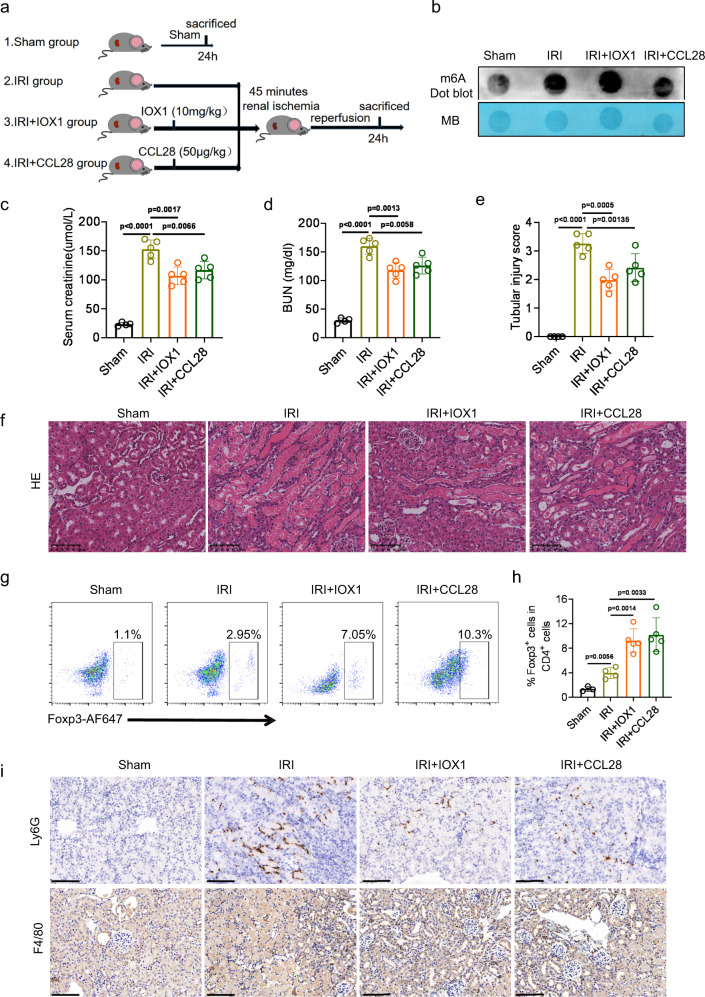
IOX1 and recombinant mouse CCL28 exerts protective effects in vivo during IRI. **a** Schematic illustrating experiment groups and treatment in 4 groups. **b** m6A dot blot assessed m6A mRNA methylation under IOX1 or CCL28 treatment in I/R-induced AKI mouse model. **c**, **d** Serum creatinine and BUN concentrations. **e**, **f** Tubular injury score and representative H&E staining image in different groups of renal tissues. **g**, **h** Representative flow cytometry dot plots and percentage of CD4^+^Foxp3^+^ cells among the CD4^+^ T cells in IRI mice with or without IOX1 and CCL28 antibody treatment. **i** Infiltration of Ly6G^+^ neutrophil and F4/80^+^ macrophages in different groups of renal tissues (**f**, **i**: Magnification: ×200, Scale bar: 100 μm). For **c**–**e** and **i**, *n* = 4, 5, 5, 5 for Sham, IRI, IRI + IOX1, IRI + CCL28 groups respectively. For **h**, *n* = 3, 4, 5, 5 for Sham, IRI, IRI + IOX1, IRI + CCL28 groups respectively. Each data point represents one animal, with unpaired two-tailed Student’s t-test performed. Data are shown as means ± SD.

IOX1 was found to be an inhibitor of ALKBH5 in previous studies^26,27^. We tested the effect of IOX1 and CCL28 in an IRI model (Fig. 8a). The results showed that the serum creatinine and BUN gradually decreased with the increase of IOX1 concentration and reached the lowest point at the 10 mg/kg concentration (Fig. S9a, S9b). The pathological damage also exhibited similar changes (Fig. S9c, S9d). Therefore, we chose 10 mg/kg as the most appropriate IOX1 concentration. IOX1 administration effectively increased m6A methylation, but no significant difference was observed in the CCL28-treated IRI group (Fig. 8b). Serum creatinine and BUN concentrations after I/R can be reduced by IOX1 or CCL28 treatment (Fig. 8c, d). H&E staining data demonstrated that IOX1 or CCL28 treatment attenuated I/R-induced renal injury (Fig. 8e, f).

Next, we detected the percentage of Tregs, neutrophils, and macrophages in kidney tissues. Increasing recruitment of Tregs was found in IOX1 and CCL28 treatment groups (Fig. 8g, h). Correspondingly, the numbers of neutrophils and macrophages decreased following IOX1 or CCL28 treatment compared to untreated IRI mice (Fig. 8i).

## Discussion

In the present study, we report the function of ALKBH5 and m6A methylation in a mouse IRI model. We found the decreased expression of *Alkbh5* after 24 h IRI treatment and *Alkbh5* deficiency in mice could protect the kidney from I/R-induced renal malfunction and proximal tubule damage. Downregulation of ALKBH5 effectively inhibited inflammation and injury by promoting CCL28 level through increased *Ccl28* mRNA m6A methylation and improved mRNA stability. The upregulation of CCL28 could recruit more Tregs and consequently inhibit the inflammation response after IRI. These results collectively showed that the ALKBH5/CCL28/Treg axis is a promising therapeutic target for renal IRI.

Recently, ALKBH5-induced m6A demethylation was found to play crucial roles in numerous diseases, especially cancer and sepsis^28–32^. Hu et al. reported that ALKBH5 regulated PKMYT1 to suppress invasion of gastric cancer via an m6A-IGF2BP3-dependent manner^30^. Another study revealed that ALKBH5 inactivated STAT3 pathway by increasing SOCS3 expression via an m6A-YTHDF2-dependent manner and affected the proliferation and tumorigenicity of osteosarcoma^33^. Also in controlling bacterial infections, Liu et al. revealed that ALKBH5-mediated m6A demethylation empowered neutrophils with high migration capability through altering the RNA decay^32^. However, studies focusing on ALKBH5 and m6A modifications in renal diseases are limited, especially for kidney IRI. Xu et al. reported differences of m6A modification and METTL14 expression in kidney biopsies of AKI patients and IRI mice, identifying YAP1 as the downstream target of METTL14^19^. Meng et al. confirmed that METTL3 contributes to renal IRI by regulating Foxd1 methylation in rats; however, the detailed mechanism was unclear^20^. Wang et al. also studied the role of METTL3 in renal IRI and other types of AKI. They found inhibition of METTL3 effectively attenuated renal injury and inflammation induced by I/R and identified TAB3 mRNA as the direct m6A modification target of METTL3^34^. These studies suggested that m6A modification is involved in renal IRI regulation but this warrants further study.

We report that the m6A demethylase ALKBH5 plays a vital role in renal IRI. Previous studies have shown an increase in the m6A modification level at 24 h and 48 h after renal IRI^19,20^. However, in our study, we observed a decrease in the level of m6A modification and an increase in the ALKBH5 level 12 h after IRI. We speculated that the increase in ALKBH5 at 12 h was mainly caused by cells other than RTECs (Fig. S1c). We have not identified these cells, and the mechanism requires further study.

In our IRI model, *Alkbh5*-KO mice showed milder kidney injury than WT mice after IRI. Conversely, *Alkbh5*-KI mice showed more severe kidney injury compared to WT mice after IRI. ALKBH5 seemed to play a harmful role in our mouse IRI model. The ALKBH5 inhibitor IOX1 could effectively protect cardiac injury by inhibiting ALKBH5 in an AMI model^27^. Here, we also showed that IOX1 could ameliorate kidney injury and inflammation induced by renal IRI, indicating that ALKBH5 is a potential target of renal IRI therapy. Its inhibitor IOX1 also warrants further research into its potential clinical applications.

However, previous studies indicating that high m6A methylation may negatively affect renal injury, we found that an *Alkbh5* knockdown-induced high m6a methylation level protects renal function. In fact, various m6A-related methylases and demethylases regulate different genes even under the same physiological and pathological conditions^15^. For example, Li et al. showed that METTL3 increased m6A methylation in glioblastoma and promoted tumor growth and progression by decreasing the m6A modification levels of serine- and arginine-rich splicing factors^35^. However, Zhang et al. found that ALKBH5 decreased m6A methylation and also promoted GBM growth by sustaining FOXM1 expression and cell proliferation^36^. The key target genes of different m6A-related methylase/demethylase may result in different outcomes.

We performed MeRIP-seq and RNA-seq combined analysis to ascertain the mechanism. Several enriched pathways were screened out through KEGG and GO analyses of differentially expressed genes and genes with different m6A peaks. Inflammatory response is known to play a vital role in AKI, and several experimental studies have demonstrated that I/R-induced AKI was abrogated when inflammation was blocked^8,37,38^. Recent studies have identified an important role of ALKBH5 in inflammation and immune cell regulation^39–41^. In our MeRIP-seq and RNA-seq analysis, several inflammation- and immune-related pathways (the TNF signaling, IL-17 signaling, cytokine-cytokine receptor interaction, neutrophil chemotaxis, and TGF-beta signaling pathways) were significantly changed, suggesting that ALKBH5 may regulate inflammation and immune response in IRI. Further analysis identified CCL28 is not only an inflammation and immune response-related chemokine, but also the candidate target with the greatest fold change difference among hypermethylation genes.

CCL28 is commonly known as mucosae-associated epithelial chemokine involved in mucosal immunity^42^. Previous studies found that hypoxia induces CCL28 expression^24,43,44^. For example, Facciabene et al. showed that tumor hypoxia induced the expression of CCL28 and that CCL28 expression correlates significantly with HIF1a expression in ovarian cancer samples^24^. In our IRI mouse model, we also found that CCL28 expression was upregulated after IRI, which possibly induced by hypoxia. In addition, the CCL28 was significantly increased in Alkbh5-KO mice compared with that in WT mice after IRI. Furthermore, we found that ALKBH5 regulates m6A modification and CCL28 expression. Mechanistically, m6A demethylation of ALKBH5 has been shown to affect mRNA stability and IGF2BP2 was involved in it^45,46^. The regulation of *Ccl28* mRNA stability was demonstrated by RT-qPCR at various time points after IRI and the interaction site in the *Ccl28* mRNA was identified using a dual-luciferase reporter assay. Therefore, we hypothesized that hypoxia and ALKBH5 together regulate CCL28 expression. In other words, hypoxia induced the expression of *Ccl28* mRNA, whose m6A modification was enhanced after ALKBH5 inhibition and stabilized by IGF2BP2. Finally, the CCL28 protein level was significantly increased.

However, CCL28 is also reported to recruit Tregs under several conditions. One study showed that hypoxia induced the expression of CCL28 in ovarian cancer cells, recruiting Tregs into the tumor^24^. Another study showed CCL28 is produced at the onset of spinal cord injury, recruiting CCR10-expressing and immunosuppressive Tregs to promote locomotor recovery^47^. Under similar conditions of hypoxia and injury, we also observed a close relationship between high CCL28 expression and the infiltration of Tregs after IRI. In other words, AKI induced CCL28 expression and subsequently contributed to the recruitment of Tregs. Tregs have been proven importance in kidney IRI and several approaches to increase Tregs have been proven to ameliorate IRI^48–50^. Tregs have an important role in suppressing innate immunity in kidney ischemia-reperfusion injury^49^. Conversely, Tregs repletion by PC61 significantly attenuated IRI-induced renal injury and leukocyte (neutrophil and macrophage) accumulation^49^. Thus, we also found that the CCL28/Treg/inflammatory cell axis is involved in the mechanism underlying the therapeutic effect of *Alkbh5* deficiency in kidney IRI.

**Fig. 9:**
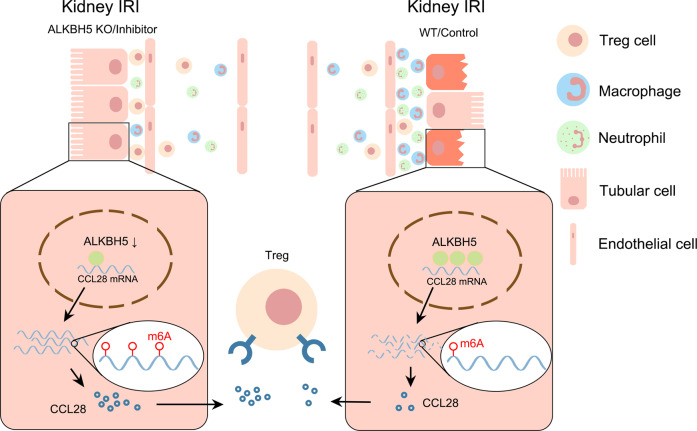
Proposed model for this study. Inhibition of ALKBH5 protects I/R-induced kidney injury through increasing CCL28 level by m6A modification, and regulating downstream CCL28/Treg/inflammatory cells axis.

To summarize, our study showed that *Alkbh5* deficiency upregulated the level of CCL28 by increasing *Ccl28* mRNA m6A methylation, thereby increasing *Ccl28* mRNA stability. Tregs were recruited through the increased CCL28 level, protecting the kidney from inhibiting inflammatory cell infiltration (Fig. 9). Our work will enrich the current knowledge of the poorly understood molecular mechanisms of m6A methylation and ALKBH5 in AKI. We also uncovered the regulation of the ALKBH5/CCL28/Treg axis in I/R-induced AKI. Overall, our findings will hopefully provide a potential strategy to treat I/R-induced AKI.

## Methods

### Animals

Male C57BL/6 mice were purchased from SLAC Laboratory Animal Co, Ltd (Shanghai, China). *Alkbh5*-knockin (KO), *Alkbh5*-knockin (KI), *Alkbh5*^flox/flox^ (*Alkbh5*^fl/fl^), and Kidney-specific promoter (cadherin-16) driven Cre (*Ksp*^Cre^) mice were purchased from Gem Pharmatech (Nanjing, China). *Ksp*^Cre^ mice were crossed with *Alkbh5*^fl/fl^ mice to generate mice heterozygous for both alleles and then obtain *Alkbh5*^fl/fl^*Ksp*^Cre^ mice. Littermates were used as controls. All animals were maintained under constant humidity and temperature at standard facilities under specific pathogen-free conditions with free access to food and water. All animal experiments were performed according to the guidelines of the Care and Use of Laboratory Animals of the Laboratory Animal Ethical Commission of Fudan University and were approved by the Animal Ethical Committee of Zhongshan Hospital, Fudan University, Shanghai, China.

### Cell lines and culture conditions

The mouse renal tubular epithelial cells (mRTECs) from ATCC were cultured in 5% fetal bovine serum (FBS) containing HyClone Dulbecco’s modified Eagle’s medium (DMEM)/F12 medium in 5% CO_2_ condition. For hypoxic groups, the cells were cultured in hypoxia condition (5% CO_2_, 1% O_2_, and 94% N_2_) for 24 h followed by 6 h or 12 h of reoxygenation.

### Adenoviral (Ad) transduction

Ad-ALKBH5, Ad-Sh-ALKBH5, and the corresponding negative controls (Ad-Control, Ad-Sh-Control) were obtained from OBiO (Shanghai, China). 1 × 10^8^ pfu/ml of NC and upregulation and downregulation ALKBH5 adenovirus were quantified and diluted in serum-free DMEM. Subsequently, the adenovirus mixtures were added to the cultured plates containing mRTEC. The supernatants were discarded after 12 h and replaced with DMEM/F12 containing 10% FBS.

### Mice renal IRI

Mice (6–8 weeks, male) were subjected to unilateral renal pedicle clamping for 45 min. The animals were kept on a warm pad to maintain the constant body temperature (37 °C). Then the clamps were released for reperfusion. A sham operation was performed in a similar manner, except for clamping of the renal pedicles. Different groups of animals were euthanasia under isoflurane at 12 h, 24 h, 48 h, 120 h, and 4 weeks after ischemia. For CCL28 treatment, recombinant mouse CCL28 (50 μg/kg) were administrated through tail vein injection before surgery. For anti-CD25 treatment, PC61 mAb (10 mg/kg) was injected intraperitoneally 3 days prior to ischemia. For CCL28 antibody treatment, CCL28 antibody (100 μg/per mouse) were administrated through tail vein injection 2 h before surgery. For IOX1 (Selleck, USA) treatment, IOX1 (10 mg/kg) were administrated through tail vein injection before surgery. When determining the dosage of IOX1 in treating the IRI mice, we set a concentration gradient of 0 mg/kg, 2 mg/kg, 5 mg/kg, 10 mg/kg, 20 mg/kg to detect the most appropriate concentration of IOX1.

### Masson’s trichrome staining

Mouse kidneys were fixed in 4% paraformaldehyde and then embedded and serial sectioned at a 5-μm thickness. Masson’s trichrome staining was used to evaluate the extent of perivascular and interstitial collagen in the kidney tissues. By optical microscopy, the collagen volume fraction was verified using ImageJ software (National Institutes of Health).

### Sirius Red

The slides were first incubated with a 0.1% Sirius Red solution dissolved in acqueous saturated pic-ric acid for 1 h, and then washed in acidified water, dehydrated. Collagen and non-collagen components were red- and orange-stained, respectively.

### RNA isolation and RT-qPCR

Total RNA from murine kidney tissues and mRTECs was isolated using TRIzol reagent (Sigma, USA). The PrimeScript RT Reagent Kit (Takara, Japan) was used for reverse transcription. qPCR was performed using HieffTM qPCR SYBR Green Master Mix (Yeasen, Shanghai, China) on an ABI Prism 7900HT (Applied Biosystems, Foster City, CA, USA). mRNA expressions of target genes were normalized to β-actin and calculated by the 2^−ΔΔ^Ct method. The primers are listed in Supplement Table 1.

### Western blot

Fresh isolated mouse kidney tissues were lysed in RIPA buffer (Beyotime, China) with protease inhibitor. After centrifugation, the protein concentrations in the supernatants were determined with a BCA protein assay kit (Beyotime, China). Protein (50 μg) were separated by SDS-PAGE on a 10% or 12% gel and then transferred to PVDF membranes. Membranes were blocked with 5% non-fat milk and incubated overnight at 4 °C with Abs against ALKBH5 (Abcam, ab195377, 1:1000), CCL28 (Abcam, ab231557, 1:1000), β-actin (Abclonal, AC026, 1:5000), METTL3 (Abclonal, A8370, 1:1000), METTL14 (Abclonal, A8530, 1:1000), WTAP (Abclonal, A22750, 1:1000), IGF2BP2 (Abclonal, A2749, 1:1000), or FTO (Abclonal, A20992, 1:1000). After washing, the membranes were incubated for 1 h at room temperature with secondary Ab. Finally, the blots were captured using ECL and analyzed by Image J (V1.8.0).

### Renal histology, immunohistochemistry, and immunofluorescent staining

Kidney sections harvested from different groups of mice were transferred to 4% paraformaldehyde and prepared by a routine procedure including fixation, dehydration, waxing, and embedding. Periodic Acid–Schiff (PAS) staining and Hematoxylin and Eosin (H&E) staining were performed, and histologic examinations were observed using light microscopy. The percentage of tubules that displayed cellular necrosis and loss of brush border were counted as follows: 0, none; 1, 0–10%; 2, 11–25%; 3, 26–45%; 4, 46–75%; 5, >75%. Immunohistochemical staining was performed according to the manufacturer’s instructions. The antibodies specific for ALKBH5 (Abcam, ab195377, 1:100), CCL28 (Abcam, ab231557, 1:100), F4/80 (Abcam, ab111101, 1:100), and Ly6G (Abcam, ab238132, 1:100) were incubated at 4 °C overnight, and the secondary antibodies were incubated for 1 h at room temperature. After staining with diaminobenzidine (DAB) and counterstaining with hematoxylin, the sections were visualized using a microscope (Olympus IX83, Japan).

### Flow cytometry of kidney-infiltrating immune cells

Kidneys were excised from mice and then incubated with complete RPMI medium plus collagenase IV (1 mg/ml, Sigma-Aldrich) and DNase I (50 μg/ml, Sigma-Aldrich) for 30 min with shaking every 10 min. The suspensions were filtered through a 70-μm filter and resuspended in FACS staining buffer. Then the cells were labeled with the appropriate combinations of Abs against cell surface markers. For intracellular protein staining, cells were fixed, permeabilized, and stained with the appropriate Abs. Flow cytometry was performed using a BD FACSAria™ III (Becton Dickinson, Franklin Lakes, NJ), and the results were analyzed with FlowJo 8.7 software. The following anti-mouse Abs were used for flow cytometry: CD45-FITC (BD biosciences, 1076109) (Biolegend, B343469), CD4-PE-Cy7 (BD biosciences, 318731), Foxp3-AF647 (BD biosciences, 1094042) (Biolegend, B343830).

### ELISA

CCL28 in lysates of mouse kidney and serum was measured by CCL28 ELISA Kit (Jiangsu Meimian Industrial Co., Ltd, China) according to the manufacturer’s instructions. Protein concentration of tissue lysates was quantified by BCA Protein Assay Reagent (Beyotime).

### Dot blot

Total RNA was isolated using TRIzol, and 2 μL RNA solution (100 ng/μL) was spotted onto a nylon membrane (Sigma-Aldrich, USA). Then, the membranes were ultraviolet crosslinked and blocked in 5% BSA for 1 h, then were incubated with rabbit anti-m6A antibody (Abcam, ab284130, 1:5000) overnight at 4 °C. After washing, the membranes were incubated with the secondary antibody (Beyotime, China) for 2 h at room temperature. Signals were detected with an enhanced chemiluminescence reagent (Millipore, USA). Then the membranes were stained with 0.02% methylene blue in 0.3 M sodium acetate (pH 5.2) for 2 h and washed to show the amount of total RNA level.

### Transwell assay

The mouse periphery blood mononuclear cells (PBMC) were isolated via a Ficoll -Paque Plus (GE Healthcare) gradient, then washed and resuspended in PBS. mRTECs (Ad-Sh-ALKBH5, Ad-Sh-Control) after 24 h hypoxia treatment were plated onto the lower compartment of 5-μm pore transwell chambers (Corning, USA). Two million fresh mice PBMCs were seeded in PBS containing 1% FBS in the upper compartment of transwell chambers. After 6 h incubation, the migrated cells were collected and used for FACS analysis of Tregs. In some experiments, 2 µg/ml CCL28 antibody were added onto the lower compartment to against CCL28.

### Measurement of ALKBH5 mRNA stability

mRTECs were transfected with Ad-Sh-Control and Ad-Sh-ALKBH5 for 48 h and treated with actinomycin D (5 μg/mL, Sigma-Aldrich) for 0, 2, 4, and 6 h. qPCR were performed to determine *Ccl28* mRNA levels. The *Ccl28* mRNA levels at 0 h were used as control.

### Dual-luciferase reporter assay

mRTECs were seeded in 24-well plates, and cultured overnight. The CCL28 pGL3-Empty and pGL3-5’UTR, pGL3-3’UTR, pGL3-CDS plasmids were individually co-transfected with either the Ad-Control or Ad-ALKBH5 and Renilla luciferase plasmids into mRTECs using Lipofectamine 3000 reagent (L3000015, Invitrogen). For next assay, pGL3-Empty, pGL3-CCL28-WT, pGL3-CCL28-Mut1, pGL3-CCL28-Mut2, pGL3-CCL28-Mut3, and pGL3-CCL28-Mut4 together with Ad-Control or Ad-ALKBH5 and Renilla luciferase plasmid were co-transfected into mTEC cells by Lipofectamine 3000 reagent (Invitrogen, USA). Then the cells were harvested 48 h after transfection for luciferease detection. Firefly luciferase activities were calculated with a Dual-Luciferase Reporter Assay System E1910 (Promega, USA) and normalized to control Renilla luciferase levels.

### Methylated RNA immunoprecipitation (MeRIP)

The MeRIP assay was conducted using a Magna RIP RNA-binding protein immunoprecipitation kit (Millipore, Billerica, MA, USA). Briefly, kidney tissues were harvested from *Alkbh5*^fl/fl^ and *Alkbh5*^fl/fl^*Ksp*^Cre^ IRI mice and total RNA was extracted. One-tenth of the RNA was saved as the input control. Magnetic beads were incubated with anti-m6A antibody (Millipore, Temecula, CA, USA) or IgG antibody at 4 °C for 4 h with rotation. After three washes, beads and RIP immunoprecipitation buffer and fragmented RNAs were mixed and incubated at 4 °C overnight with rotation. After proteinase K buffer digestion, methylated mRNAs were purified with alcohol for qPCR to detect the enrichment of *Ccl28* mRNA.

### RNA immunoprecipitation (RIP)

RIP was conducted using the EZ-Magna RIP RNA-Binding Protein Immunoprecipitation Kit (Merck Millipore, USA) according to the manufacturer’s instructions. In brief, cells were harvested and lysed in complete RIP lysis buffer. Then the cell extracts were incubated with IP buffer containing magnetic beads coated with anti-mouse IgG (Merck Millipore) or anti-FLAG (CST) overnight. Subsequently, the RNA-protein complexes were washed and incubated with proteinase K. Total RNA was then isolated from the extracts using TRIzol reagent and analyzed by qPCR.

### Methylated RIP-sequencing (MeRIP-seq) and RNA-sequencing (RNA-seq)

MeRIP-seq and RNA-seq were performed by OE Biotech Co., Ltd. (Shanghai, China). In brief, total RNA from WT and *Alkbh5*-KO IRI kidney were isolated and extracted with TRIzol reagent (Invitrogen) following the manufacturer’s procedure. About 25 μg of total RNA was chemically fragmented 150-nt-long oligonucleotides using divalent cations under elevated temperature. Approximately ^1^/_10_ of the fragmented RNA was separated as input control for further RNA-seq. The remaining fragmented RNA was mixed with 50 μl Protein A premixed with 16 g anti-m6A antibody at 4 °C overnight in IP buffer [50 mM Tris-HCl, 750 mM NaCl and 0.5% Igepal CA-630]. Eluted RNA was precipitated by 75% ethanol. Eluted m6A-containing fragments (IP) and untreated input control fragments are converted to final cDNA library in accordance with a strand-specific library preparation by dUTP method. The average insert size for the paired-end libraries was ~150 bp. And the paired-end 2 × 150 bp sequencing were performed on an Illumina NovaSeq 6000 platform. m6A-enriched peaks in each m6A IP sample were identified using MeTDiff peak calling software (version 1.1.0) and the corresponding input sample served as a control. Differentially methylated peaks between groups were detected using MeTDiff with parameter and annotated using ChIPseeker (version 1.12.1). GO enrichment and KEGG pathway enrichment analyses of the identified peaks and differential peaks were performed using R based on hypergeometric distribution. Sequence motifs were identified using MEME and DREME and annotated using Tomtom software from MEME suite (version 5.0.5). For RNA-Seq, differential expressed genes (mRNA, lncRNA, and circRNA) were categorized as upregulation and downregulation. In MeRIP-Seq, the differential regulation of gene methylation was noted according to the changes in peak abundance. Python (v.2.7.12) script was used for the correlation analyses of the two omics contents and to simultaneously compare transcription and methylation levels.

### Statistical analysis

Data were expressed as the mean ± SD of at least three biological replicates. The differences between two groups were assessed using Student’s t-test for normally distributed data. *P* < 0.05 indicated significance. All statistical analysis was performed using GraphPad Prism 9.

### Reporting summary

Further information on research design is available in the Nature Portfolio Reporting Summary linked to this article.

## Supplementary information

Supplementary Information

Reporting Summary

## Data Availability

MeRIP-seq and RNA-seq datasets generated as part of this study can be downloaded from the GEO database with the accession code: GSE224289. The authors declare that all other data supporting the findings of this study are available within the article and its Supplementary Information files, or are available from the authors upon request. Source data are provided with this paper.

## Source data

Source Data
