# Supplementary material for: Inhibition of ALKBH5 attenuates I/R-induced renal injury in male mice by promoting Ccl28 m6A modification and increasing Treg recruitment
Source: Nat Commun. 2023 Mar 1;14:1161. doi: 10.1038/s41467-023-36747-y (PMC9977869; doi:10.1038/s41467-023-36747-y)
Supplement: Supplementary file 1 — Supplementary Information [file 41467_2023_36747_MOESM1_ESM.pdf]

Inhibition of ALKBH5 attenuates I/R-induced renal injury in male mice by promoting *Ccl28* m6A modification and increasing Treg recruitment

Juntao Chen, Cuidi Xu, Kun Yang, Rifeng Gao, Yirui Cao, Lifei Liang, Siyue Chen, Shihao Xu, Ruiming Rong, Jina Wang, Tongyu Zhu

Supplementary Figure 1.

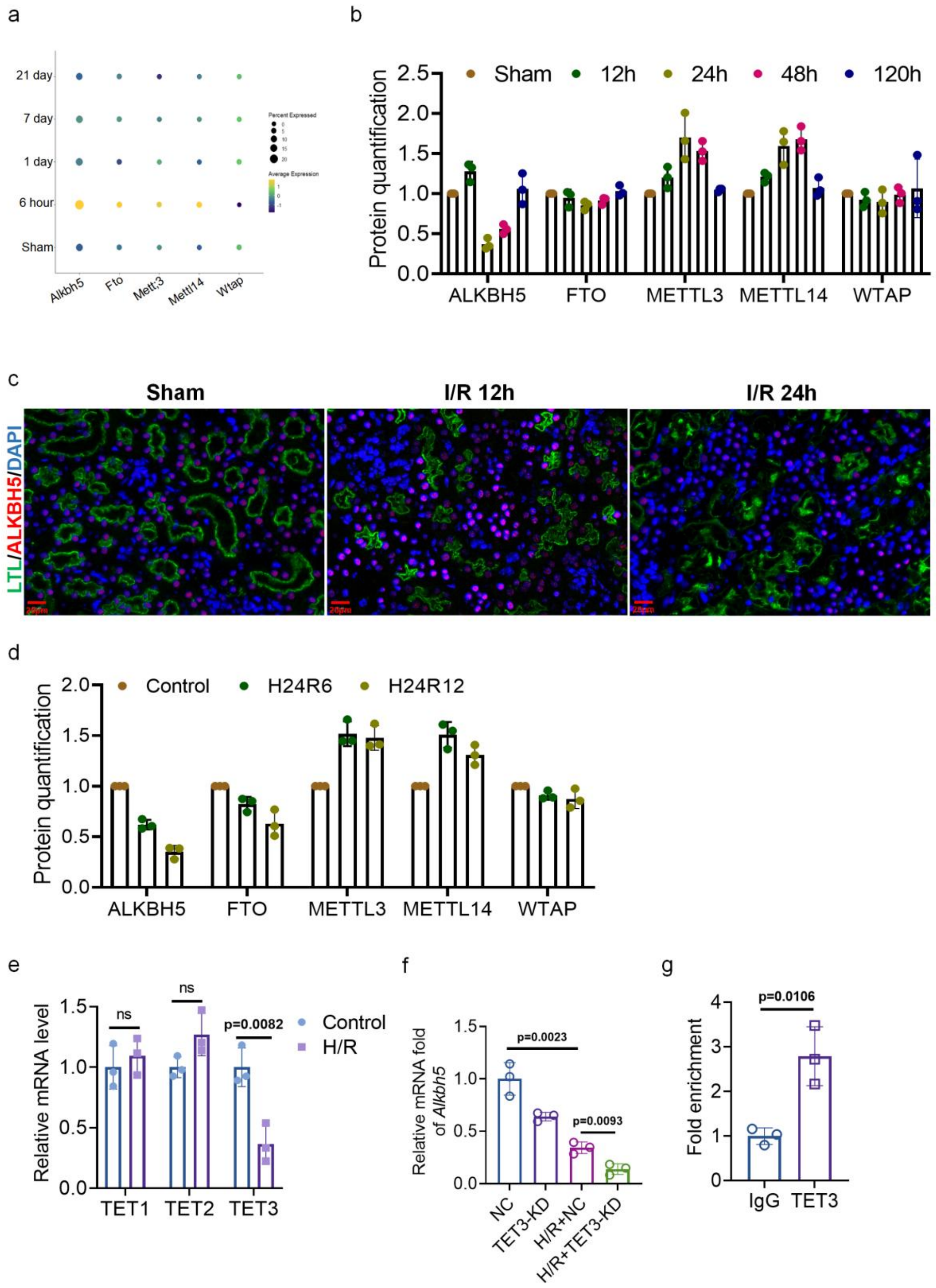

The m6A modification and ALKBH5 were involved in renal I/R and the H/R response in TECs. (a) The analyse of m6A modification related genes in different time (Sham, 6 hour, 1 day, 7 day, 21 day) after mouse IRI on a Mouse IRI Kidney single-cell sequencing from GEO database. (b) Western blot analysis of ALKBH5, FTO, METTL3, METTL14 and WTAP in kidneys at different time post-IRI. (c) Representative IF staining of ALKBH5 (red), LTL (green) and DAPI (blue) in kidney biopsies from sham, I/R 12h, and I/R 24h mice. (Magnification: ×400, Scale bar: 20μm). (n = 5 mice per group) (d) Western blot analysis of ALKBH5, FTO, METTL3, METTL14 and WTAP in mRTECs. (e) RNA level of TETs in control or H/R groups was examined by RT-qPCR. (f) RNA level of *Alkbh5* in NC or H/R groups with/without TET3-KD was examined by RT-qPCR. (g) ChIP assay using anti-TET3 or IgG in mRTEC cells and RT-qPCR of the ALKBH5 promoter. Values correspond to the ratio between the anti-TET3 antibody immunoprecipitated DNA relative to the IgG immunoprecipitated DNA. For b, d-g, n=3 biologically independent samples/experiments. Data are presented as means ± SD. Unpaired two-tailed Student's t-test.

Supplementary Figure 2.

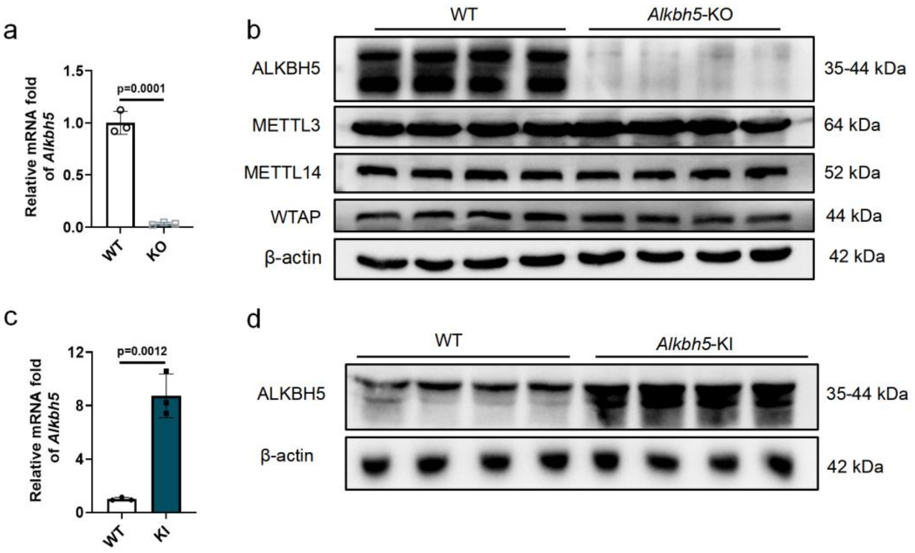

The *Alkbh5* was successful knockout or knockin in *Alkbh5*-KO or *Alkbh5*-KI mice. (a) Knockout of *Alkbh5* was confirmed by RT-qPCR. (b) Western blot analysis of ALKBH5, METTL3, METTL14 and WTAP in KO and WT mice kidney. (c and d) Knockin of *Alkbh5* was confirmed by RT-qPCR and Western blot analysis. For a, c, n=3 biologically independent animals. Data are presented as means ± SD.

Supplementary Figure 3.

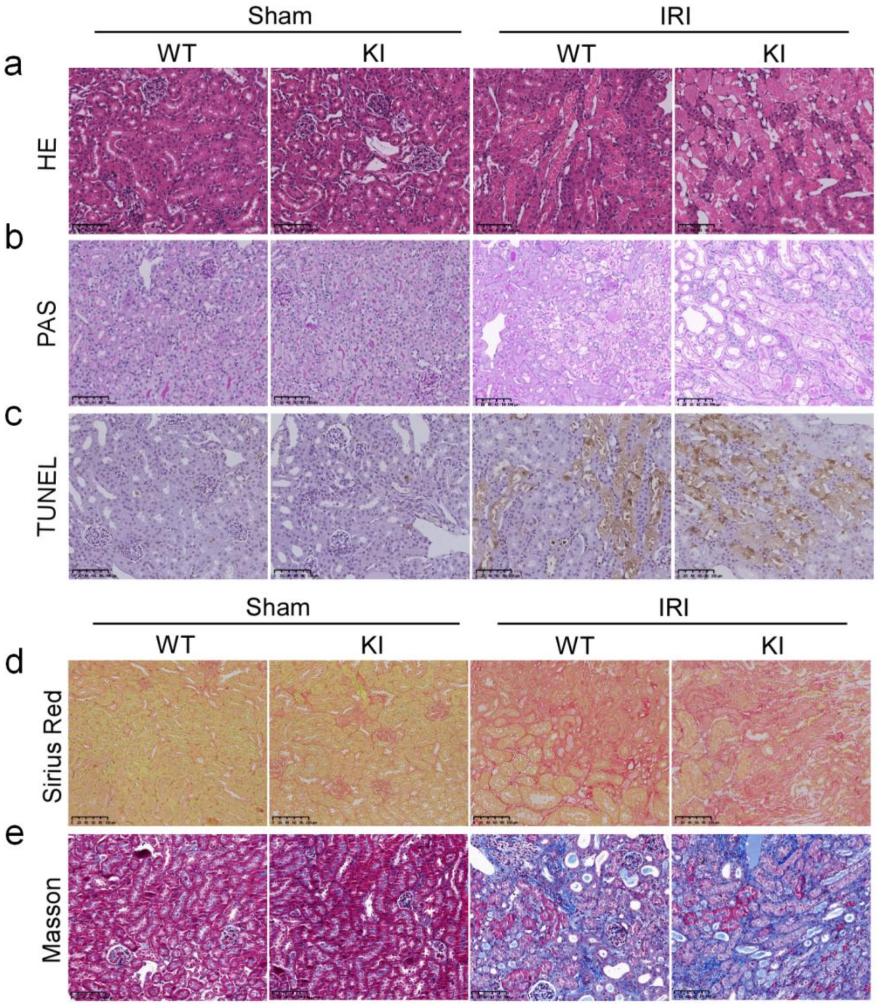

***Alkbh5* overexpression altered I/R-induced acute kidney injury and fibrosis.** (a and b) Representative H&E and PAS staining image in different groups of renal tissues. (c) The apoptosis levels in different groups were detected by TUNEL staining. (d and e) Sirius red and masson staining in different groups of IRI mouse model. (Magnification: ×200, Scale bar: 100μm). (a-e: n=5 for all groups)

.

Supplementary Figure 4.

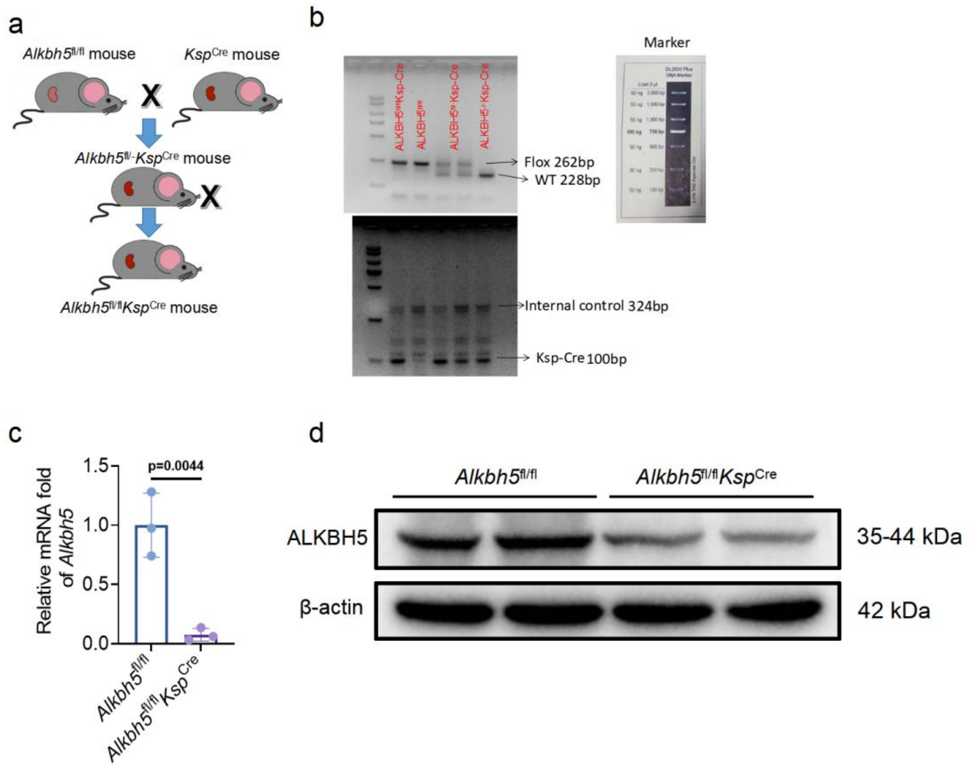

The cKO mice were crossed from *Alkbh5*$^{\mathbf{fl/fl}}$ mince and *Ksp*^{Cre} mice. (a) Schematic illustrating the genetic approach used to generate *Alkbh5* cKO mice. (b) *Alkbh5* deficiency was confirmed by assessing genomic DNA. (c and d). *Alkbh5* deficiency was confirmed by RT-qPCR and Western blot analysis. For c, n=3 biologically independent animals. Data are presented as means ± SD.

Supplementary Figure 5.

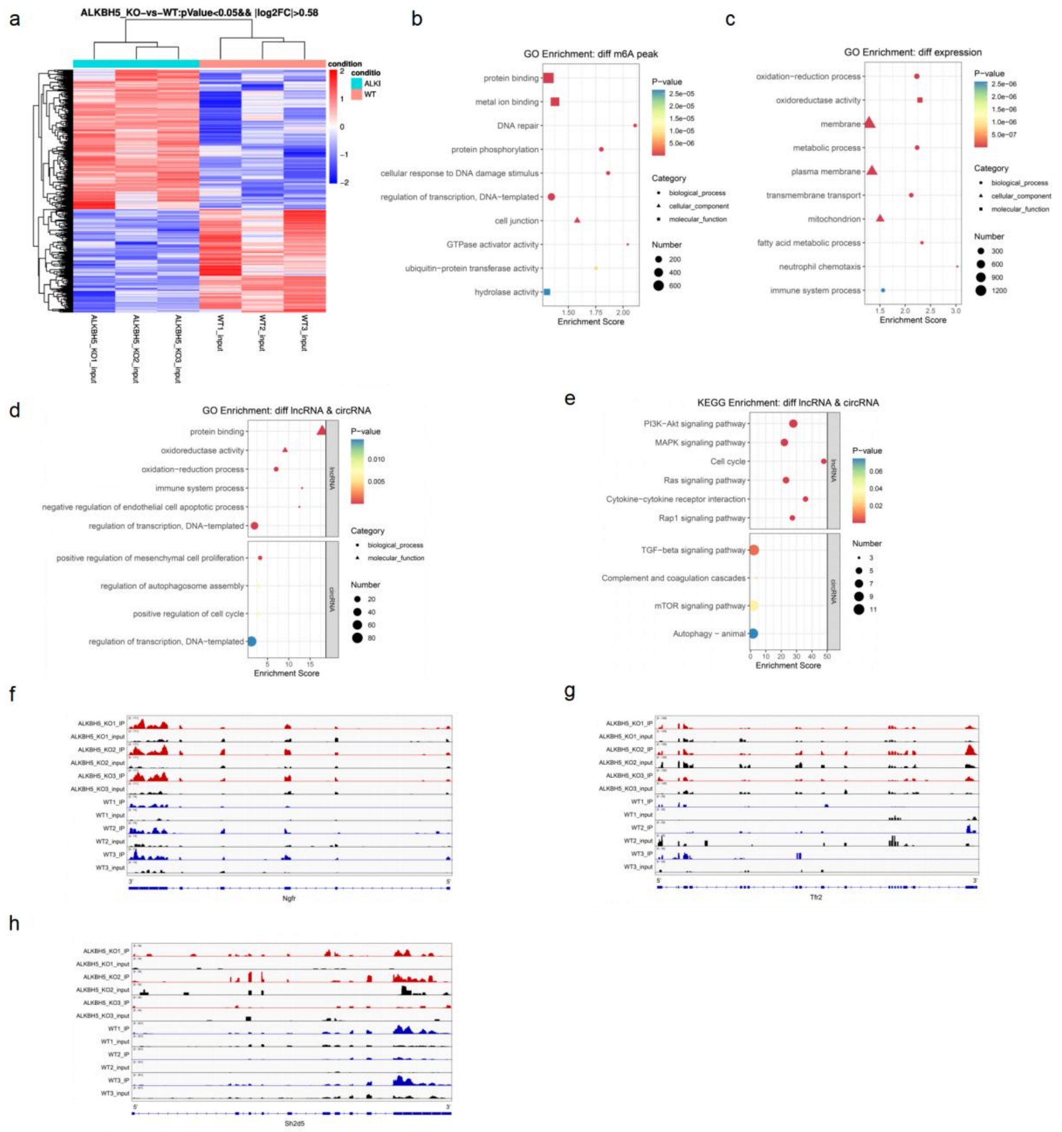

Characterization of m6A Modification and Gene Expression Changes in the *Alkbh5*-KO mice. (a) Heatmap of all differentially expressed genes. (b-c) GO enrichment analysis correlated with different expression genes and different m6A peak genes. (d) GO enrichment analysis correlated with different expression lncRNA and circRNA. (e) KEGG enrichment analysis correlated with different expression lncRNA and circRNA. (f-h) Deficiency of ALKBH5 increased m6A modification of *Ngfr*, *Tfr2* and *Sh2d5* mRNA in IRI-KO group. Differential expression analysis was performed using the DESeq2 R package based on negative binomial distribution model. GO and KEGG enrichment p-value was calculated based on the hypergeometric distribution.

Supplementary Figure 6.

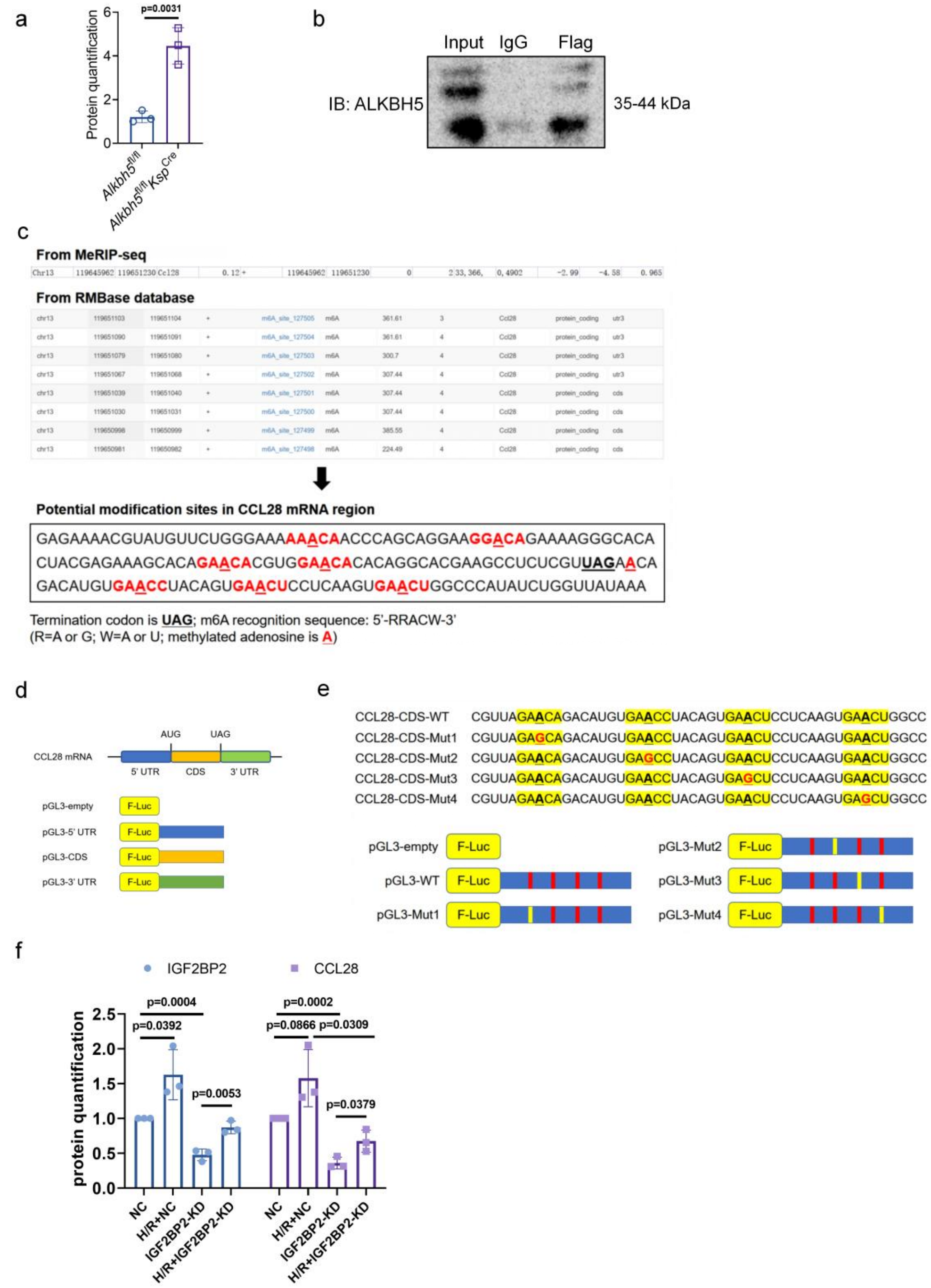

CCL28 is a direct target of ALKBH5. (a) Western blot analysis of CCL28 in different groups. n=3 biologically independent animals. (b) Immunoblotting of ALKBH5 in IgG and Flag group. Three biological repeated immunoblots have been performed. (c) Potential modification sites in *Ccl28* mRNA region. (d) Schematic illustrating of the position of luciferase reporter. (e) Schematic illustrating of the design of the mutation of luciferase reporter. (f) Western blot analysis of IGF2BP2 and CCL28 in different groups. n=3 biologically independent experiments. Data are

presented as means ± SD.

Supplementary Figure 7.

a

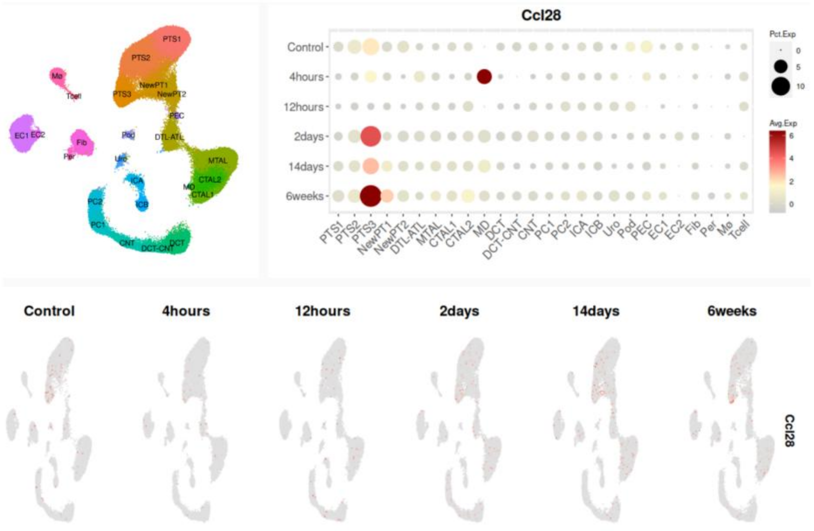

b

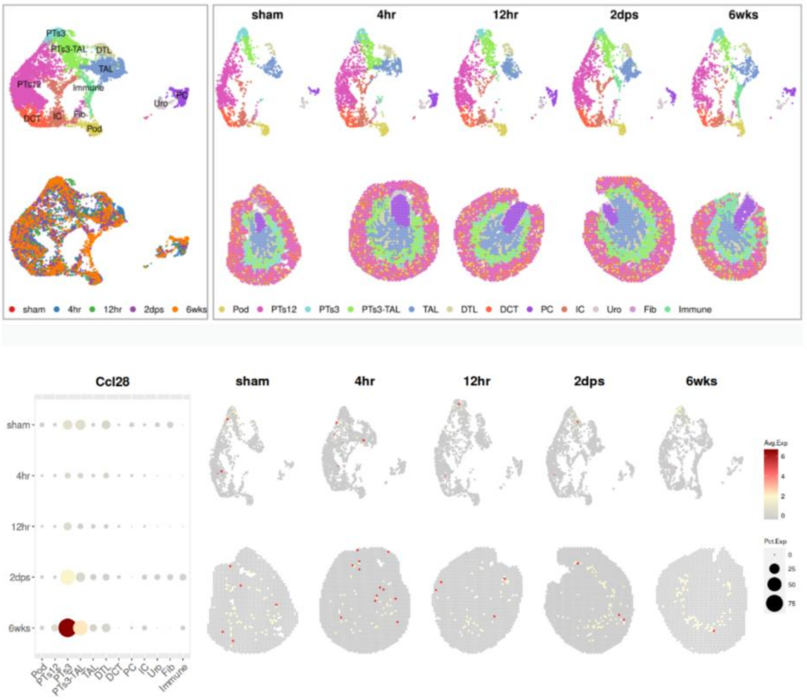

***Ccl28* increased after IRI.** (a) A single cell sequencing of Mouse IRI Kidney from Kidney Interactive Transcriptomics (an online analyzer for kidney single cell datasets, http://humphreyslab.com/SingleCell/) showed the expression change of *Ccl28* after IRI. (b) A Spatial Transcriptomics of Female Mouse IRI Kidney from Kidney Interactive Transcriptomics showed the expression change of *Ccl28* after IRI.

Supplementary Figure 8.

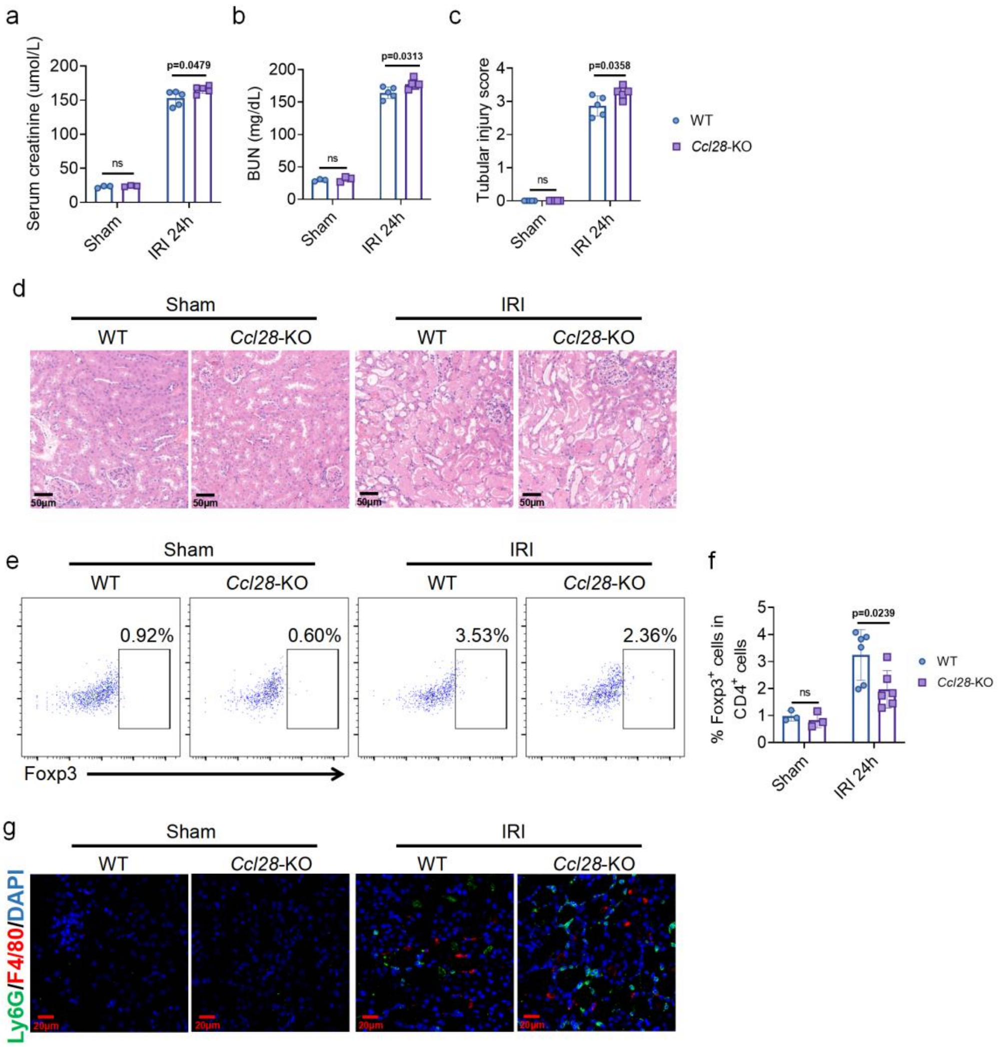

CCL28 protected I/R-induced acute kidney injury. (a and b) Serum creatinine and BUN concentrations. (c and d) Representative H&E staining image and tubular injury score in different groups of renal tissues. (d: Magnification: ×200*,* Scale bar: 50μm). (e and f) Representative flow cytometry dot plots and percentage of $CD4^{+}FOXP3^{+}$ cells among the $CD4^{+}$ T cells in different groups. (g) Representative IF staining of F4/80 (red), Ly6G (green) and DAPI (blue) in kidney from different group mice. (Magnification: ×400, Scale bar: 20μm). For a, b, and g n=3 for Sham groups, n=5 for IRI 24h groups. For c, n=5 for all groups. For f, n=3 for Sham groups, n=6 for IRI 24h groups. Each data point represent one animal, with unpaired two-tailed Student's t-test performed. Data are presented as means ± SD.

Supplementary Figure 9.

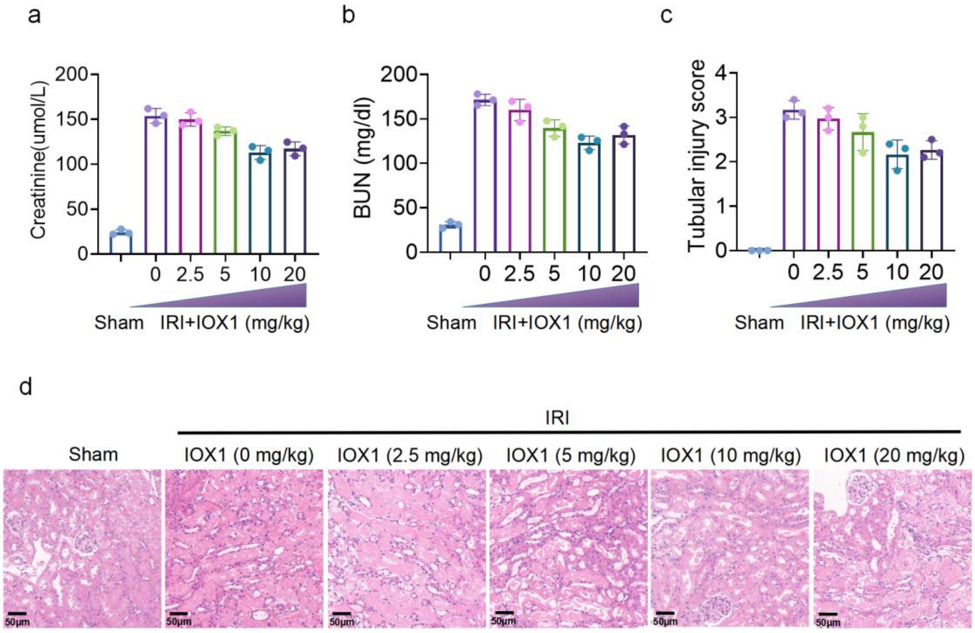

IOX1 on Different concentrations have different effect on protecting I/R induced acute kidney injury. (a and b) Serum creatinine and BUN concentrations. (c and d) representative H&E staining image and tubular injury score in different groups of renal tissues. (d: Magnification: ×200, Scale bar: 50μm). For a-c, n=3. Each data point represent one animal.

Supplementary Table 1. The qRT-PCR primers

Gene	Forward 5'-3'	Reverse 5'-3'
ALKBH5	TGCTGCGTATGGGGCTTAAA	ATGCCTAACAGGAGCAACCC
CCL28	CATACTTCCCATGGCCTCC	GAGAGGCTTCGTGCCTGTG
FTO	TCACAGACGTGGTTTCCGAG	ACCACTGGGTTGAGAGGAGT
METTL3	CCCAACCTTCCGTAGTGATAG	TGGCGTAGAGATGGCAAGAC
METTL14	GGTCGGAGTGTGAACCTGAT	GGTCCTCTTCCACGCTGTAT
WTAP	TAATGGCGAAGTGTCGAATG	CTGCTGTCGTGTCTCCTTCA
β-actin	ATGACCCAAGCCGAGAAGG	TGCAATGACGTGAGGAACACT
TET1	TCTGTTGTTGTGCCTCTGGA	GCCTTTAAAACTTTGGGCTTC
TET2	GAGACGCTGAGGAAATACGG	TGGTGCCATAAGAGTGGACA
TET3	CCCACAAGGACCAGCATAAC	CCATCTTGTACAGGGGGAGA
