# Supplementary material for: Inhibition of ALKBH5 attenuates I/R-induced renal injury in male mice by promoting Ccl28 m6A modification and increasing Treg recruitment
Source: Nat Commun. 2023 Mar 1;14:1161. doi: 10.1038/s41467-023-36747-y (PMC9977869; doi:10.1038/s41467-023-36747-y)
Supplement: Supplementary file 2 — Reporting Summary [file 41467_2023_36747_MOESM2_ESM.pdf]

nature portfolio

Corresponding author(s): Tongyu Zhu, Jina Wang, Ruiming Rong

Last updated by author(s): Jan 30, 2023

# Reporting Summary

Nature Portfolio wishes to improve the reproducibility of the work that we publish. This form provides structure for consistency and transparency in reporting. For further information on Nature Portfolio policies, see our Editorial Policies and the Editorial Policy Checklist.

## Statistics

For all statistical analyses, confirm that the following items are present in the figure legend, table legend, main text, or Methods section.

| n/a | Confirmed | |
|---|---|---|
| ☐ | ☒ | The exact sample size (*n*) for each experimental group/condition, given as a discrete number and unit of measurement |
| ☐ | ☒ | A statement on whether measurements were taken from distinct samples or whether the same sample was measured repeatedly |
| ☐ | ☒ | The statistical test(s) used AND whether they are one- or two-sided<br>*Only common tests should be described solely by name; describe more complex techniques in the Methods section.* |
| ☒ | ☐ | A description of all covariates tested |
| ☐ | ☒ | A description of any assumptions or corrections, such as tests of normality and adjustment for multiple comparisons |
| ☐ | ☒ | A full description of the statistical parameters including central tendency (e.g. means) or other basic estimates (e.g. regression coefficient) AND variation (e.g. standard deviation) or associated estimates of uncertainty (e.g. confidence intervals) |
| ☐ | ☒ | For null hypothesis testing, the test statistic (e.g. *F*, *t*, *r*) with confidence intervals, effect sizes, degrees of freedom and *P* value noted<br>*Give P values as exact values whenever suitable.* |
| ☒ | ☐ | For Bayesian analysis, information on the choice of priors and Markov chain Monte Carlo settings |
| ☒ | ☐ | For hierarchical and complex designs, identification of the appropriate level for tests and full reporting of outcomes |
| ☒ | ☐ | Estimates of effect sizes (e.g. Cohen's *d*, Pearson's *r*), indicating how they were calculated |

*Our web collection on statistics for biologists contains articles on many of the points above.*

## Software and code

Policy information about availability of computer code

| | |
|---|---|
| Data collection | sequencing data were produced by illumina Novaseq 6000 |
| Data analysis | For MeRIP-Seq: m6A-enriched peaks in each m6A IP sample were identified using MeTDiff peak calling software (version1.1.0).<br>Differentially methylated peaks between groups were detected using MeTDiff with parameter and annotated using ChIPseeker(version1.12.1).<br>MEME and DREME from MEME suite (version5.0.5) were used to detect the motif sequence, Tomtom software from MEME suite (version5.0.5) was used to annotate the motif .<br>Python (v.2.7.12) script was used for the correlation analyses of the two omics contents and to simultaneously compare transcription and methylation levels<br>Numerical computing and statistical analysis were conducted using Graphpad 9.0.<br>Western Blot was analyzed by Image J (V1.8.0)<br>the flow cytometry results were analyzed with FlowJo 8.7 software |

For manuscripts utilizing custom algorithms or software that are central to the research but not yet described in published literature, software must be made available to editors and reviewers. We strongly encourage code deposition in a community repository (e.g. GitHub). See the Nature Portfolio guidelines for submitting code & software for further information.

## Data

Policy information about availability of data

All manuscripts must include a data availability statement. This statement should provide the following information, where applicable:

- Accession codes, unique identifiers, or web links for publicly available datasets
- A description of any restrictions on data availability
- For clinical datasets or third party data, please ensure that the statement adheres to our policy

The MeRIP-seq data as part of this study can be downloaded from GEO ( we have deposited the data used in our paper into GEO database , but we are still waiting for the relevant accession codes now).The authors declare that all other data supporting the findings of this study are available within the article and its Supplementary information files, or are avilable from the authors upon request. The source date are provided as a Source Data file.

## Human research participants

Policy information about studies involving human research participants and Sex and Gender in Research.

| | |
|---|---|
| Reporting on sex and gender | N/A |
| Population characteristics | N/A |
| Recruitment | N/A |
| Ethics oversight | N/A |

Note that full information on the approval of the study protocol must also be provided in the manuscript.

# Field-specific reporting

Please select the one below that is the best fit for your research. If you are not sure, read the appropriate sections before making your selection.

☒ Life sciences ☐ Behavioural & social sciences ☐ Ecological, evolutionary & environmental sciences

For a reference copy of the document with all sections, see nature.com/documents/nr-reporting-summary-flat.pdf

# Life sciences study design

All studies must disclose on these points even when the disclosure is negative.

| | |
|---|---|
| Sample size | No statistical method was used to predetermine samle size. Sample sizes were chosen based on those used in previous and preliminary studies and preliminary studies from our lab which allow for statistical analysis, Sample sizes are indicated in the Figure, Figure legends or main text |
| Data exclusions | No data were excluded from the study |
| Replication | Numbers of replicates were started in the figure legends and Method section |
| Randomization | Male C57BL/6 mice, at 6-8 weeks of age, were randomly assigned to sham operation or I/R model using a single sequence of simple randomization assignments. No randomization was done for the other experiments |
| Blinding | investigators were blinded to group allocation during data collection and analysis |

# Reporting for specific materials, systems and methods

We require information from authors about some types of materials, experimental systems and methods used in many studies. Here, indicate whether each material, system or method listed is relevant to your study. If you are not sure if a list item applies to your research, read the appropriate section before selecting a response.

## Materials & experimental systems

| n/a | Involved in the study | |
|---|---|---|
| ☐ | ☒ | Antibodies |
| ☐ | ☒ | Eukaryotic cell lines |
| ☒ | ☐ | Palaeontology and archaeology |
| ☐ | ☒ | Animals and other organisms |
| ☒ | ☐ | Clinical data |
| ☒ | ☐ | Dual use research of concern |

## Methods

| n/a | Involved in the study | |
|---|---|---|
| ☒ | ☐ | ChIP-seq |
| ☐ | ☒ | Flow cytometry |
| ☒ | ☐ | MRI-based neuroimaging |

# Antibodies

Antibodies used

ALKBH5 (Abcam, ab195377, WB:1:1000, IF: 1:100),
CCL28 (Abcam, ab231557, WB: 1:1000, IHC: 1:100),
β-actin (Abclonal, AC026, WB: 1:5000),
METTL3 (Abclonal, A8370, WB: 1:1000),
METTL14 (Abclonal, A8530, WB: 1:1000),
WTAP (Abclonal, A22750, WB: 1:1000),
IGF2BP2 (Abclonal, A2749, WB: 1:1000),
FTO (Abclonal, A20992, WB: 1:1000),
m6A (Abcam, ab284130, Dot blot: 1:5000)
F4/80 (Abcam, ab111101, IF: 1:100) ,
Ly6G (Abcam, ab238132, IF: 1:100),
CD45 (BD biosciences, 1076109) (Biolegend, B343469),
CD4 (BD biosciences, 318731),
Foxp3 (BD biosciences, 1094042) (Biolegend, B343830)

Validation

All the commercially available antibodies were validated by the manufacturer via immunoblot or IF imaging.

# Eukaryotic cell lines

Policy information about cell lines and Sex and Gender in Research

Cell line source(s)

mRTEC cells, (purchased from National Collection of Authenticated Cell Cultures)

Authentication

The cell line used in this study was authenticated using STR profiling

Mycoplasma contamination

Yes-comfirmed Mycoplasma negative by National Collection of Authenticated Cell Cultures for validation studies,

Commonly misidentified lines
(See ICLAC register)

None

# Animals and other research organisms

Policy information about studies involving animals; ARRIVE guidelines recommended for reporting animal research, and Sex and Gender in Research

Laboratory animals

Male C57BL/6 mice were purchased from SLAC Laboratory Animal Co, Ltd (Shanghai, China). Alkbh5-knockin (KO), Alkbh5-knockin (KI), Alkbh5flox/flox (Alkbh5fl/fl) and Kidney-specific promoter (cadherin-16) driven Cre (KspCre) mice were purchased from Gem Pharmatech (Nanjing, China). KspCre mice were crossed with Alkbh5fl/fl mice to generate mice heterozygous for both alleles and then obtain Alkbh5fl/flKspCre mice. Littermates were used as controls. All animals were maintained under constant humidity and temperature at standard facilities under specific pathogen-free conditions with free access to food and water.

Wild animals

the study did not involved in wild animals

Reporting on sex

The male mice were used for this study

Field-collected samples

No field-collected samples were used in this study

Ethics oversight

All experimental procedures were approved by the Animal Care Ethics Committee of the Zhongshan Hospital, Fudan University and were performed in accordance withe the National Institutes of Health Guide for the Care and Use of Laboratory Animals

Note that full information on the approval of the study protocol must also be provided in the manuscript.

# Flow Cytometry

## Plots

Confirm that:

- [x] The axis labels state the marker and fluorochrome used (e.g. CD4-FITC).
- [x] The axis scales are clearly visible. Include numbers along axes only for bottom left plot of group (a 'group' is an analysis of identical markers).
- [x] All plots are contour plots with outliers or pseudocolor plots.
- [x] A numerical value for number of cells or percentage (with statistics) is provided.

## Methodology

| | |
|---|---|
| Sample preparation | Kidneys were excised from mice and then incubated with complete RPMI medium plus collagenase IV (1 mg/ml, Sigma-Aldrich) and DNase I (50µg/ml, Sigma-Aldrich) for 30 min with shaking every 10 min. The suspensions were filtered through a 70-µm filter and resuspended in FACS staining buffer. |
| Instrument | BD FACSAria™ III (Becton Dickinson, Franklin Lakes, NJ) |
| Software | the results were analyzed with FlowJo 8.7 software |
| Cell population abundance | the cell population abundance was determined by cell counting |
| Gating strategy | based on the size and intracellular condition of the lymphocyte and the experiments, we identified the FSC/SSC gates of lymphocytes. Then the CD45, CD4, and FOXP3 gate were identified basing on compensated monopositive tube control |

- [x] Tick this box to confirm that a figure exemplifying the gating strategy is provided in the Supplementary Information.
